# The established chemokine-related prognostic gene signature in prostate cancer: Implications for anti-androgen and immunotherapies

**DOI:** 10.3389/fimmu.2022.1009634

**Published:** 2022-10-06

**Authors:** Lei Chen, Yi Zheng, Changqin Jiang, Cheng Yang, Li Zhang, Chaozhao Liang

**Affiliations:** ^1^ Department of Urology, The First Affiliated Hospital of Anhui Medical University, Hefei, China; ^2^ Institute of Urology, Anhui Medical University, Hefei, China; ^3^ Anhui Province Key Laboratory of Genitourinary Diseases, Anhui Medical University, Hefei, China

**Keywords:** chemokine, prognosis, gene signature, androgen deprivation therapy, immunotherapy, prostate cancer

## Abstract

**Background:**

Prostate cancer (PCa) was one of the most common malignancies among men, while the prognosis for PCa patients was poor, especially for patients with recurrent and advanced diseases.

**Materials and methods:**

Five PCa cohorts were downloaded from The Cancer Genome Atlas and Gene Expression Omnibus databases, and the biochemical recurrence (BCR)-related chemokine genes were identified by LASSO-Cox regression. The chemokine-related prognostic gene signature (CRPGS) was established, and its association with PCa patients’ clinical, pathological and immune characteristics was analyzed. The association between CRPGS and PCa patients’ responses to androgen deprivation therapy (ADT) and immunotherapy was analyzed. The CRPGS was compared with other previously published molecular signatures, and the CRPGS was externally validated in our real-world AHMU-PC cohort.

**Results:**

Four recurrence-free survival (RFS)-related chemokine genes (CXCL14, CCL20, CCL24, and CCL26) were identified, and the CRPGS was established based on the four identified chemokine genes, and TCGA-PRAD patients with high riskscores exhibited poorer RFS, which was validated in the GSE70768 cohort. The CRPGS was associated with the clinical, pathological, and immune characteristics of PCa patients. Low-risk PCa patients were predicted to respond better to ADT and immunotherapy. By comparing with other molecular signatures, the CRPGS could classify PCa patients into two risk groups well, and the CRPGS was associated with the m6A level, as well as TP53 and SPOP mutation status of PCa patients. In the AHMU-PC cohort, the CRPGS was associated with the advanced pathology stage and Gleason score.

**Conclusions:**

The identified chemokine genes and CRPGS were associated with the prognosis of PCa, which could predict PCa patients’ responses to anti-androgen and immunotherapies.

## Introduction

As one of the most common cancer types in older males, there will be 268, 490 new prostate cancer (PCa) cases and 34, 500 deaths in the United States in 2022, which accounts for 27% and 11% of the top ten leading cancer types, respectively (1). In recent years, the promotion of PSA-based screening has led to an increase in PCa incidence, and early detection decreased PCa-specified mortality (2). With the advance in PCa treatment, including radical prostatectomy (RP), chemotherapy, androgen deprivation therapy (ADT), and radiotherapy, patients’ prognosis has improved greatly (3, 4). However, the prognosis for PCa patients with metastatic and castration-resistant diseases was poor, and novel approaches were explored to improve patients’ outcomes, including androgen receptor signaling inhibitor (ARSI) (5), and immunotherapy (6, 7). However, metastatic and castration-resistant PCa patients’ prognoses remained unsatisfactory.

Chemokines (also known as chemotactic cytokines) belong to the small secreted protein superfamily with chemotactic activity to induce cell migration, and chemokines act as the ligand to bind their receptors. There are four types of chemokines (C, CC, CXC, and CX3C), and most chemokines exerted their effects by interacting with their 7-transmember G-protein coupled receptors (8). CXCL3 and CXCL5 were detected to be overexpressed in PCa tissues and cells, and overexpression of CXCL3 and CXCL5 promoted the growth of PCa cells *via* the autocrine or paracrine pathway (9, 10). Chemokines also participated in the recruitment of immune cells into the tumor microenvironment (TME) (11). For example, CCL2 enhanced the angiogenesis and metastasis of PCa, and CCL2 affected macrophage infiltration in PCa tissue (12). CXCL12/CXCR4 axis exerted crucial roles in the progression of tumors. CXCL12 enhanced the migration of PCa cells, and inhibition of CXCR4 reversed the effects, and PCa patients with bone metastasis exhibited a higher positive rate of CXCR4 protein than bone metastasis negative patients (13). The CXCR7/RDC1 axis was identified as the downstream of the CXCL12/CXCR4 axis, which was associated with the aggressiveness of PCa (14), and antibody against CXCL12 inhibited the proliferation of LNCaP C4-2B and PC3 cells (15), implying the potential therapeutic value of the CXCL12/CXCR4 axis in PCa. Moreover, targeting chemokines became a promising approach for cancer therapy, including skin and lung cancer, etc. However, the therapeutic value of chemokines has not been applied in PCa, and more research investigating the diagnostic, prognostic, and therapeutic roles of chemokines in PCa is warranted.

In the current study, we established the chemokine-related prognostic gene signature (CRPGS) based on the chemokine genes, and the CRPGS was associated with the clinical, pathological, and immune features of PCa patients. The association between CRPGS and patients’ responses to ADT and immunotherapy was investigated, and we also compared CRPGS with other previously established molecular signatures, and the CRPGS-based nomogram was constructed to better apply the CRPGS into practice. Finally, the CRPGS was validated by our real-world Anhui Medical University-Prostate Cancer (AHMU-PC) cohort.

## Materials and method

### Data processing

**Figure 1:**
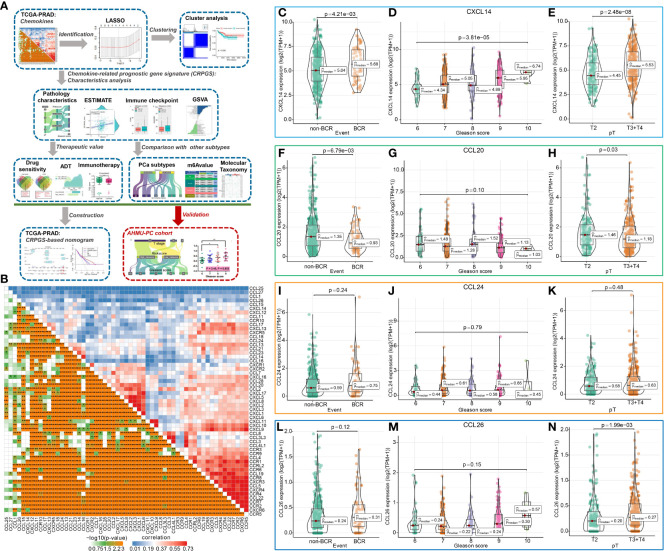
The chemokine-related prognostic genes in PCa. The whole process was shown in the flowchart **(A)**. The association among 57chemokine genes in PCa **(B)**, and the distribution of CXCL14, CCL20, CCL24, and CCL26 expression levels in PCa patients with different BCR statuses, Gleason scores, and pathology T stages **(C–N)**.

The flowchart showed the procedures of the current study (**Figure 1A**). The Cancer Genome Atlas-Prostate Adenocarcinoma (TCGA-PRAD) RNA-Seq data and the corresponding clinical data were downloaded from the TCGA database, and the GSE70768, GSE70769, GSE46602, and GSE150368 cohorts were downloaded from the Gene Expression Omnibus (GEO) database. Additionally, 69 pathologically confirmed PCa patients in our real-world AHMU-PC cohort were included, and the pathology and clinical information of these 69 PCa patients were detailedly described in our previous study (16). This study was approved by the ethics committee of the First Affiliated Hospital of Anhui Medical University (PJ 2022-09-22).

### Establishment of chemokine-related prognostic gene signature (CRPGS) in PCa

Univariate Cox regression analysis and LASSO-Cox regression analysis were performed to identify prognostic chemokine genes, and the CRPGS was established based on the identified chemokine genes, and the detailed information could be found in the Supplementary Materials and Methods.

### Gene set variation analysis (GSVA)

GSVA was performed to explore the alterations in CRPGS-related pathway activity of PCa (17), and to quantify the androgen receptor (AR) signaling activity, which was detailedly described in the **Supplementary Materials and Methods.**

### Consensus cluster analysis

The consensus cluster analysis was performed to investigate the role of the four identified chemokine genes in PCa, and detailed information could be found in the **Supplementary Materials and Methods.**

### The association between CRPGS and PCa patients’ clinical and immune characteristics

The association between riskscore and chemokine gene expression levels, age, BCR status, Gleason score, tumor T and N stage, residual tumor status and immune infiltration level, immunophenoscore (IPS) (18), and CYT score (19) was analyzed, and the detailed information could be found in the Supplementary Materials and Methods.

### The identified chemokine genes and drug response

The effects of the identified chemokine genes on PCa patients’ responses to drug therapy were predicted by the Computational Analysis of REsistance (CARE) (20) and CellMiner (21) databases, and the detailed information could be found in the Supplementary Materials and Methods.

### The association between CRPGS, androgen deprivation therapy (ADT), and immunotherapy

Based on the important roles of ADT in PCa treatment, we investigated the association between CRPGS and ADT response by quantifying AR signaling activity and estimating the IC50 of bicalutamide. Additionally, we analyzed the expression of the immune checkpoint between different risk groups, and the GSE78220 cohort and TIDE score were also used to investigate the association between CRPGS and immunotherapy response (22, 23), and the detailed information could be found in the **Supplementary Materials and Methods.**

### Comparison of CRPGS with other molecular signatures

The established CRPGS was compared with our two previously published PCa signatures (16, 24) and another five PCa molecular classifiers to evaluate the wide application of CRPGS (25–29), which was described in the **Supplementary Materials and Methods.**

### Establishment of the CRPGS-based nomogram for RFS prediction

The CRPGS-based nomogram was established to predict the RFS of PCa, and the detailed information could be found in the **Supplementary Materials and Methods.**

### Validation of the CRPGS in real-world AHMU-PC cohort

The real-world AHMU-PC cohort included 69 pathologically confirmed PCa patients, and the PCa tissues were collected and RNA-sequence was performed, which was described in our previous study (16). The gene expression level was transformed into log2(TPM+1). The distribution of CXCL14, CCL20, CCL24, CCL26, and riskscore was compared between different pathologic groups.

### Statistically analysis

For continuous variables, the *t*-test or Wilcoxon test was used to compare the difference between two groups, and one-way ANOVA or Kruskal−Wallis was used to compare the difference among more than two groups. For categorical variables, χ^2^ test was used to examine the differences between groups. Survival analysis was performed based on Kaplan-Meier and log-rank tests. and *P* (two sides) < 0.05 was considered statistically significant. All the statistical procedures were performed using the R software.

## Results

### Identification of chemokine-related prognostic genes in PCa

The flowchart showed the study procedures (**Figure 1A**). In total, 57 genes of CXCL, CCL, and their receptors were extracted from the TCGA-PRAD cohort, and the enrichment analysis indicated that the 57 chemokine genes were mainly enriched in cell chemotaxis, chemokine-mediated signaling pathway, T cell migration, etc. (**Supplemental Figures 1A, B**). The correlation plot showed the association among these 57 genes with Pearson coefficients (**Figure 1B**), and **Supplemental Figure 1C** displayed the interaction among these 57 genes, which was divided into two models by Molecular COmplex Detection (MCODE). Then, the LASSO-Cox regression identified four BCR-related chemokine genes, including CXCL14, CCL20, CCL24, and CCL26 (**Supplemental Figures 2A, B**), and the association among these four chemokine genes was shown in **Supplemental Figure 2C**. The expression levels of CXCL14, CCL20, and CCL26 were associated with the BCR, Gleason score, and pathology T stage of PCa (**Figures 1C–N**), and the expressions of these four genes were positively associated with the ESTIMATE score, immune score, and stromal scores of PCa (**Supplemental Figures 3A–D**).

Moreover, the expressions of CXCL14, CCL20, CCL24, and CCL26 were associated with tumor purity and infiltration of CD4+ T cell, CD8+ T cell, B cell, macrophage, dendritic cell, and neutrophil in PCa (**Supplemental Figures 4A–D**). PCa patients with somatic copy number alterations of CCL20, CCL24, and CCL26 had different levels of neutrophils and CD8+ T cells (**Supplemental Figures 5A–D**). By analyzing the single-cell RNA sequencing of human prostate tissue, we found that CXCL14, CCL20, and CCL26 were mainly expressed in smooth muscle cells, fibroblasts, urothelial cells, and basal prostatic cells, while CCL24 was not detected by the single-cell RNA sequence of the prostate tissue (**Supplemental Figures 6A–E**).

### Four identified chemokine genes classify PCa samples into two clusters

**Figure 2:**
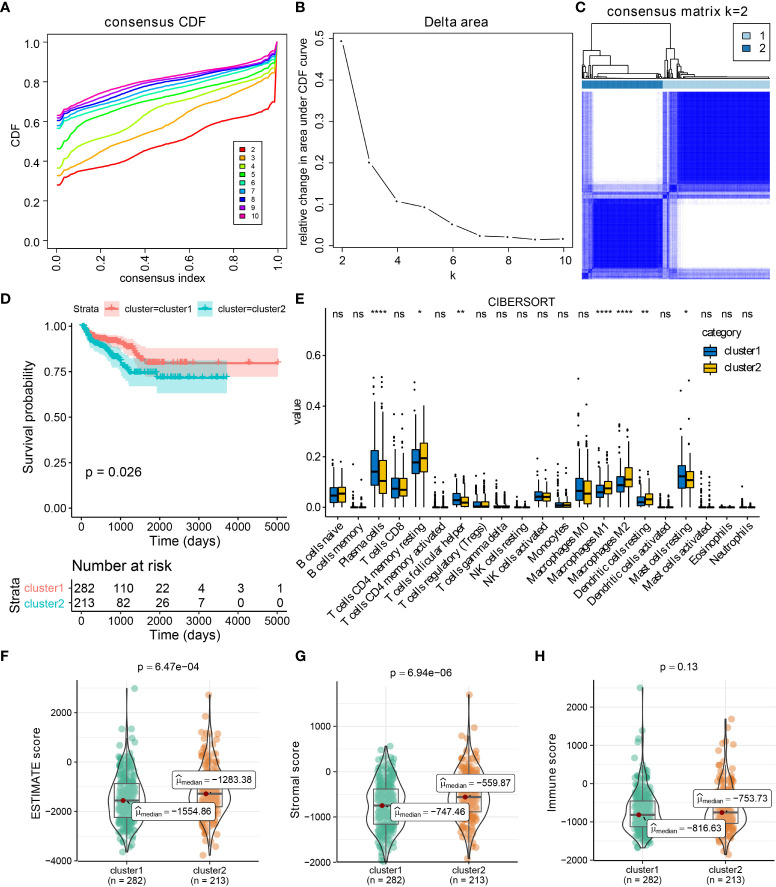
CXCL14, CCL20, CCL24, and CCL26 defined the TCGA-PRAD samples into two clusters. Results of consensus cluster analysis in TCGA-PRAD cohort based on CXCL14, CCL20, CCL24, and CCL26 expression levels **(A–C)**, and survival analysis between cluster1 and cluster2 **(D)**. The immune profiles of PCa in cluster1 and cluster2 by CIBERSORT method **(E)**. The distribution of ESTIMATE, immune, and stromal scores in PCa patients from different clusters **(F–H)**. *p < 0.05; **p < 0.01; ****p < 0.0001; ns: not significant.

By performing consensus cluster analysis, PCa patients in TCGA-PRAD were divided into two clusters based on CXCL14, CCL20, CCL24, and CCL26 (**Figures 2A–C**), and compared to patients in cluster 1, PCa patients in cluster 2 had poor RFS (**Figure 2D**). Compared to patients in cluster 2, PCa patients in cluster 1 exhibited a higher level of T cells follicular helper, plasma cells, and mast cells resting, while patients in cluster 1 had a lower level of T cells CD4 memory resting, macrophages M1 and M2, etc. (**Figure 2E**). Furthermore, compared to patients in cluster 1, PCa patients belonging to cluster 2 had higher ESTIMATE scores, and stromal scores (**Figures 2F–H**). Hence, the roles of CXCL14, CCL20, CCL24, and CCL26 in PCa deserved further exploration.

### The implications of chemokine genes for drug therapy in PCa

The expression of CXCL14 was negatively associated with the IC50 of 6-Thioguanine and 6-THIOGUANINE by CellMiner (all *P* < 0.001, **Supplemental Figures 7A, B**), and the expression levels of CCL20, CCL24, and CCL26 were positively associated with the IC50 of Rebimastat, CFI-400945, Barasertib, SNS-314, PF-04217903, SGX-523, and volitinib (all *P* < 0.001, **Supplemental Figures 7C–I**) respectively. PCa patients with a low riskscore may benefit more from CFI−400945 therapy (**Supplemental Figure 7J**). The results CARE of indicated that expression levels of CXCL14, CCL20, and CCL26 were negatively associated with CARE scores for many drugs from CCLE, GDSC/CGP, and CTRP datasets, implying their negative correlation with drug efficacy, while the expression of CCL24 was positively associated with drug efficacy (**Supplemental Figure 7K–N**). The common drugs that targeted CXCL14, CCL20, CCL24, and CCL26 were displayed in the Venn diagrams (**Supplemental Figures 7O, P**).

### Establishment and validation of chemokine-related prognostic gene signature (CRPGS) for RFS prediction of PCa

**Figure 3:**
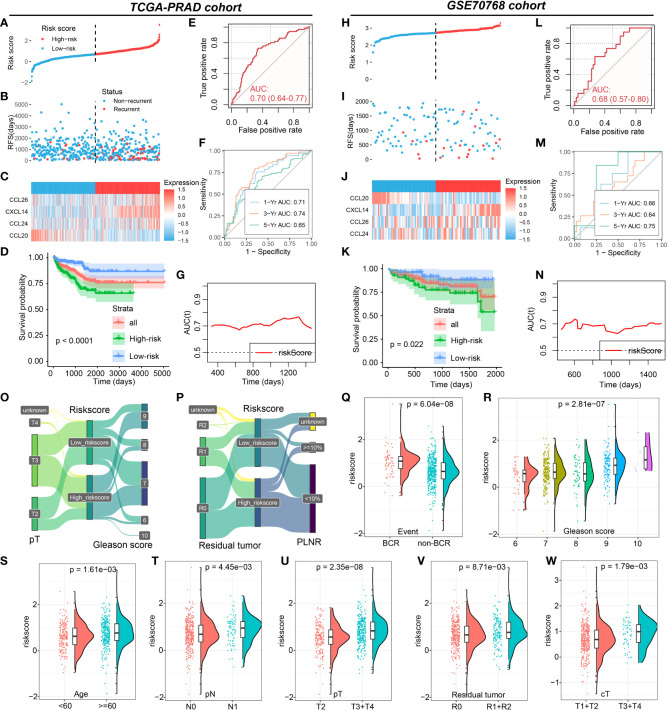
Roles of CRPGS in predicting the RFS of PCa. The distribution of the riskscore **(A, B)**, and the expression of CXCL14, CCL20, CCL24, and CCL26 in high- and low-risk patients in the TCGA-PRAD cohort **(C)**. Survival analysis between high- and low-risk PCa patients **(D)**, and the ROC, time-dependent ROC, and AUC curves curve showed the RFS predictivity of CRPGS in the TCGA-PRAD cohort **(E–G)**. The distribution of the riskscore **(H,I)**, and the expression of CXCL14, CCL20, CCL24, and CCL26 in high- and low-risk patients in the GSE70768 cohort **(J)**. Survival analysis between high- and low-risk PCa patients **(K)**, and the ROC, time-dependent ROC, and AUC curves curve showed the RFS predictivity of CRPGS in the GSE70768 cohort **(L–N)**. The distribution of T stage, Gleason score, residual tumor, and PLNR in different risk groups by the Sanky plots **(O,P)**. The distribution of riskscore in PCa patients with different BCR statuses, age, Gleason score, residual tumor, and tumor stages **(Q–W)**.

Based on the important role of CXCL14, CCL20, CCL24, and CCL26 in PCa, the CRPGS was established by using the LASSO-Cox regression, which was as follows: riskscore = (CXCL14 expression * 0.185018842290928) + [CCL20 expression * (-0.408867899574549)] + (CCL24 expression * 0.280649328317824) + (CCL26 expression * 0.33033065384401). Based on the median value of CRPGS, TCGA-PRAD patients were allocated to high- and low-risk groups. The distribution of the riskscore was displayed in **Figures 3A, B**, and the heat map showed the expression of CXCL14, CCL20, CCL24, and CCL26 in low- and high-risk patients (**Figure 3C**). Compared to patients with low riskscores, PCa patients with high riskscores had poorer RFS (**Figure 3D**), and the ROC curve showed a good predictive value of the CRPGS in RFS prediction, with an AUC of 0.70 (**Figure 3E**), and time-dependent ROC and AUC curves further demonstrated its predictive value, with a 1/3/5-year AUC of 0.71, 0.74, 0.65, respectively (**Figures 3F, G**). The GSE70768 cohort was used to validate the established CRPGS. The distribution of the riskscore was shown in **Figures 3H, I**, and the heat map indicated the expression of CXCL14, CCL20, CCL24, and CCL26 in low- and high-risk patients (**Figure 3J**), and high-risk patients had poorer RFS (**Figure 3K**). ROC and time-dependent ROC curves showed good predictivity of the CRPGS in the GSE70768 cohort, with an AUC of 0.68 and a 1/3/5-year AUC of 0.66, 0.64, 0.75, respectively (**Figures 3L, M**), and the time-dependent AUC curve further demonstrated its clinical value in RFS prediction (**Figure 3N**).

### Association between the CRPGS, immune characteristics, and immunotherapy of PCa

**Figure 4:**
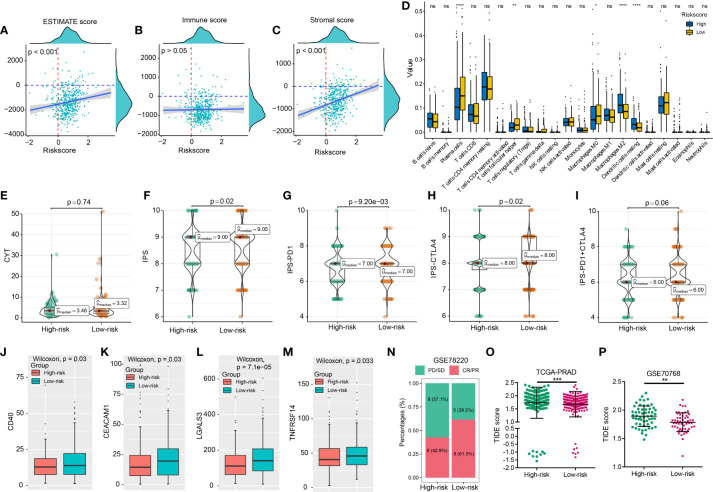
CRPGS was related to the immune characteristics of PCa. The association between riskscore and ESTIMATE, immune, and stromal scores **(A–C)**, and the immune profiles between high- and low-risk patients **(D)**. The distribution of CYT scores, IPS, IPS-PD1, IPS-CTLA4, IPS-PD1+CTLA4 scores between low- and high-risk patients **(E–I)**. The association between riskscore and the expression levels of immune checkpoints in PCa patients **(J–M)**. The histogram showed the proportion of patients’ responses in high- and low-risk groups from the GSE78220 cohort **(N)**. The distribution of TIDE scores in high- and low-risk patients in the TCGA-PRAD and GSE70768 cohort **(O, P)**. *p < 0.05; **p < 0.01; ***p < 0.001; ****p < 0.0001; ns, not significant.

PCa patients with a high riskscore had higher levels of CXCL14, CCL24, and CCL26 expression, while patients with a high riskscore exhibited lower levels of CCL20 expression (**Supplemental Figures 8A–D**), which was consistent with the coefficients of the CRPGS. The Sanky plots showed the distributions of T stage, Gleason score, residual tumor, and positive lymph node ratio (PLNR) in different risk groups (**Figures 3O, P**). PCa patients with BCR had a higher riskscore than patients without BCR, and PCa patients with advanced pathology stage had a higher riskscore than patients with early pathology stage (**Figures 3Q–W**). Moreover, riskscore was positively related to ESTIMATE score and stromal score (**Figures 4A–C**), and compared to PCa with a low riskscore, patients with high riskscores had decreased levels of plasma cells, T cells follicular helper, and macrophages M0, while had elevated levels of macrophages M2 and dendritic cells (**Figure 4D**). Additionally, no significant difference in CYT scores between low- and high-risk patients (**Figure 4E**). PCa patients with high riskscore had low IPS, IPS-PD1, and IPS-CTLA4 socres than patients with low riskscore, and no significant difference in IPS-PD1+CTLA4 score between these two groups (**Figures 4F–I**). High-risk patients had lower levels of CD40, CEACAM1, LGALS3, and TNFRSF14 than low-risk patients (**Figures 4J–M**). In GSE78220 melanoma cohorts, low-risk patients exhibited a higher proportion of PR/CR status (61.5% *vs* 42.9%, **Figure 4N**). Results of TIDE showed that PCa patients with low riskscores had lower TIDE scores (**Figures 4O, P**). Taken together, low-risk PCa patients may respond better to immunotherapy.

### Roles of CRPGS in androgen response pathway and ADT response

**Figure 5:**
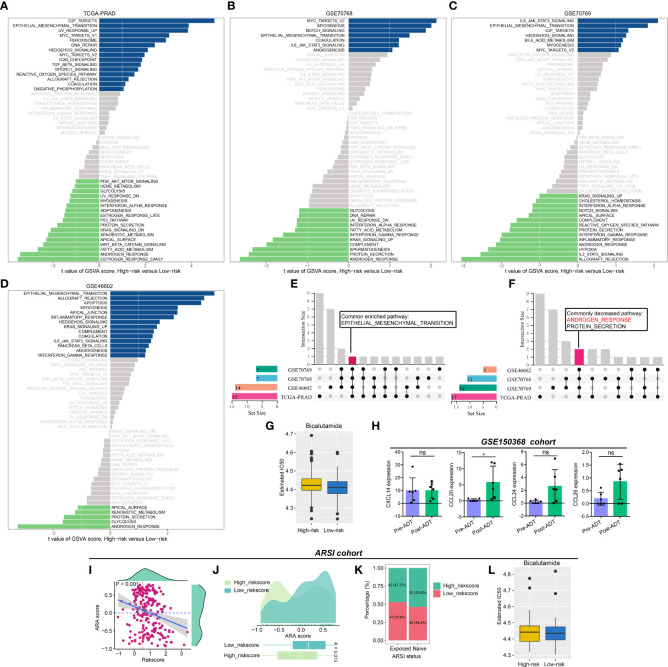
Association between CRPGS, androgen response pathway, and ADT response. Results of GSVA between high- and low-risk PCa patients **(A–D)**, and the Venn diagram showed the common enriched and decreased pathways **(E, F)**. The estimated IC50 of bicalutamide between high- and low-risk patients in TCGA-PRAD cohort **(G)**. The alterations in the expression level of CXCL14, CCL20, CCL24, and CCL26 after ADT treatment in the GSE150368 cohort **(H)**. The association between CRPGS, ARA score, and ARSI exposure status **(I–K)**, and the estimated IC50 of bicalutamide between high-and low-risk patients in Abida et al’s cohort **(L)**. *p < 0.05; ns, not significant.

We performed GSVA to evaluate the pathway activities between low- and high-risk PCa patients. As shown in **Figures 5A–D**, several pathway activities of 50 hallmark gene sets were altered in the four PCa cohorts, and the changes in the pathway activity of “ANDROGEN_RESPONSE” attracted our interest by the Venn diagram (**Figures 5E, F**). Compared to PCa patients with high riskscores, the estimated IC50 of bicalutamide was lower in low-risk patients (**Figure 5G**). Additionally, the expression level of CCL20 was increased after receiving ADT in the GSE150368 cohort (**Figure 5H**). In Abida et al’s cohort, the CRPGS was negatively associated with AR activity (ARA) score, and patients with lower riskscore exhibited higher ARA scores (**Figures 5I, J**). In PCa patients exposed to ARSI score, 52.8% of patients belong to the low-risk group, while in patients with ARSI naïve, 46.2% individuals belong to the low-risk group (**Figure 5K**), and low-risk patients exposed to ARSI had lower estimated IC50 of bicalutamide (**Figure 5L**).

### Comparison of CRPGS with other PCa molecular classifiers

**Figure 6:**
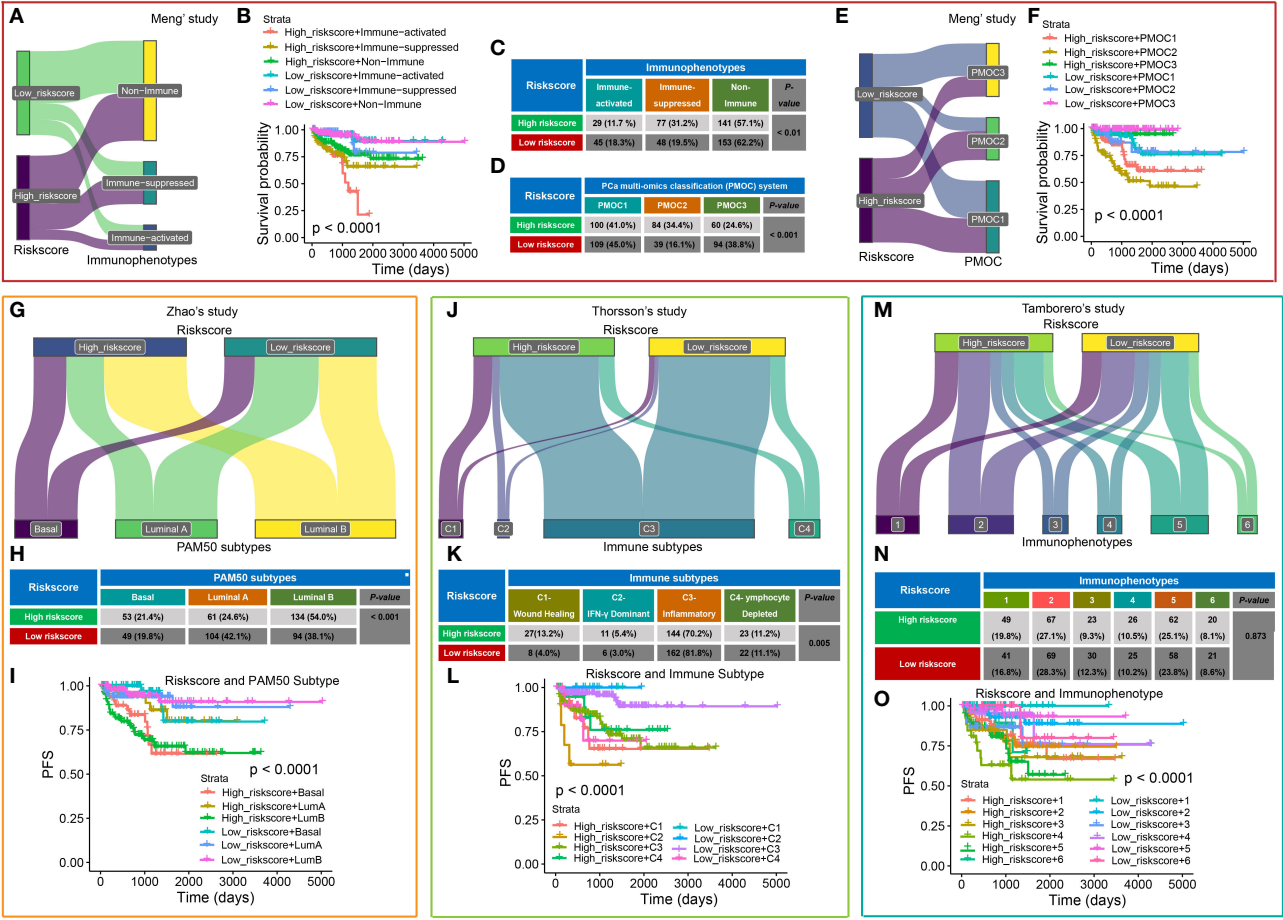
Comparison of CRPGS with other PCa molecular signatures. Sanky plot showed the comparison results of CRPGS with our previously established immune subtypes (non-immune, immune-suppressed, and immune-activated subtypes **(A)**, the combined effects of CRPGS + immune subtypes on PCa patients’ survival **(B)**, and the χ^2^ test table showed the distribution of CRPGS in different immune subtypes **(C)**. For the PMOC subtypes, the χ^2^ test table showed the distribution of CRPGS in different PMOC subtypes **(D)**, the Sanky plot showed the comparison results of CRPGS with the PMOC subtypes **(E)**, the combined effects of CRPGS + PMOC subtypes on PCa patients’ survival **(F)**. PAM50 classifier, Sanky plot showed the comparison results of CRPGS with PAM50 classifier **(G)**, and the χ^2^ test table showed the distribution of CRPGS in different PAM50 classifiers **(H)**, and the combined effects of CRPGS + PAM50 classifier on PCa patients’ survival **(I)**. For Thorsson’s study, Sanky plot showed the comparison results of CRPGS with the four immune subtypes **(J)**, and the χ^2^ test table showed the distribution of CRPGS in the four immune subtypes **(K)**, and the combined effects of CRPGS + the four immune subtypes on PCa patients’ survival **(L)**. For Tamborero’s study, Sanky plot showed the comparison results of CRPGS with the six immunophenotypes **(M)**, and the χ^2^ test table showed the distribution of CRPGS in different immunophenotypes **(N)**, and the combined effects of CRPGS + immunophenotypes on PCa patients’ survival **(O)**.

We also compared the CRPGS with our previously established two PCa-related molecular signatures (16, 24). As shown in **Figures 6A–C**, most PCa patients belong to the non-immune group, while 31.2% of high-risk patients belong to the immune-suppressed group, and 18.3% of low-risk patients belong to the immune-activated group (*P* < 0.01), and PCa patients with high riskscore + immune-activated/suppressed subtypes exhibited poorer prognosis (*P* < 0.001). Additionally, most PCa patients belong to the PMOC1 subtypes, and 34.4% of high-risk patients belong to the PMOC2 subtypes while 38.8% of low-risk patients belong to the PMOC3 subtypes (**Figures 6D–F**, *P* < 0.001), and patients with high riskscore + PMOC2 exhibited poorer outcomes, which was consistent with our previous results (24).

The CRPGS was also compared with another three published molecular subtypes. Zhao et al (26) developed a PAM50 classifier to divide the 22 carcinomas into luminal (luminal A and luminal B) and basal-like subtypes. More than half of high-risk patients belong to luminal B subtypes (54.0%), and most low-risk patients belong to luminal A (42.1%) (*P* < 0.001), and high-risk + luminal B/basal-like patients had poorer RFS (**Figures 6G–I**). Thorsson et al (28) defined six immune subtypes across 33 cancer types, and our results showed that most PCa patients belong to the inflammatory subtypes, and 13.2% of high-risk and 4.0% of low-risk patients belong to the wound healing subtype, and 5.4% of high-risk and 3.0% of low-risk patients belong to IFN-γ dominant subtype (*P* < 0.01), and patients with high-risk + C1/C2 exhibited poorer RFS (**Figures 6J–L**). Tamborero et al (27) identified six immunophenotypes across 29 solid cancers, and patients with high cytotoxic immunophenotypes tend to have better survival. Compared with Tamborero et al’s study (27), we found that PCa patients with different riskscores were almost average distributed into these six immunophenotypes (*P* = 0.873, **Figures 6M, N**), while patients with high-risk + immunophenotype 4/5 had poorer survival(*P* < 0.001, **Figure 6O**).

**Figure 7:**
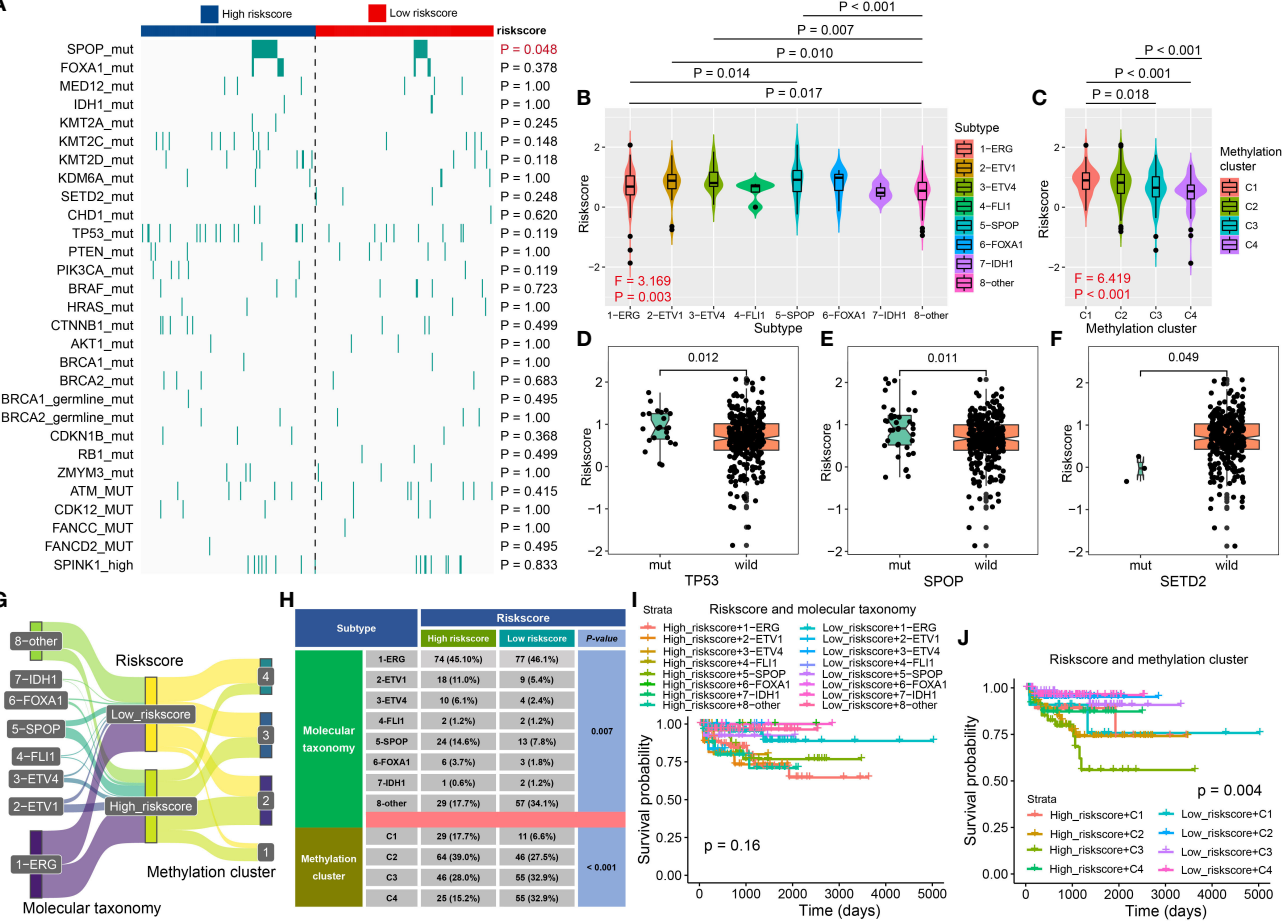
The association between CRPGS and SPOP, TP53, SETD2 mutation, and DNA methylation level. The heat map showed the distribution of SPOP mutation, FOXA1 mutation, IDH1 mutation, etc., between high- and low-risk PCa patients **(A)**. The distribution of riskscore in different molecular subtypes, DNA methylation clusters, TP53, SPOP, and SETD2 mutation **(B–F)**. Sanky plot showed the comparison results of CRPGS with molecular taxonomy **(G)**, and the χ^2^ test table showed the distribution of CRPGS in different molecular taxonomies **(H)**, and the combined effects of CRPGS + molecular taxonomy/methylation cluster on PCa patients’ survival **(I–J)**.

The Cancer Genome Atlas Research Network classified 76% of 333 primary TCGA-PCa into seven molecular subtypes (25). We revealed that high-risk PCa patients had a higher proportion of SPOP mutation (*P* = 0.048, **Figure 7A**), and the PCa patients with SPOP mutation subtype had a higher riskscore than patients with ERG fusion (*P* = 0.014, **Figure 7B**). Additionally, patients with methylation cluster C1 and C2 exhibited a higher riskscore than C3 and C4 (*P* < 0.05, **Figure 7C**) respectively. Patients with TP53 and SPOP mutation had higher riskscore than wild ones (all *P* < 0.05, **Figures 7D, E**), while patients with SETD2 mutation had lower riskscore compared to wild ones (*P* < 0.05, **Figure 7F**). For Molecular taxonomy, the Sankey plot and the contingency table showed most PCa patients belong to ERG fusion subtypes, and 14.6% of high-risk patients belong to the SPOP mutation subtype, while only 7.8% of low-risk patients belong to the SPOP mutation subtype (*P* = 0.007, **Figure 7G**). Additionally, 17.7% and 15.2% of high-risk PCa patients belong to methylation clusters C1 and C4, while 6.6% and 32.9% of low-risk patients belong to clusters C1 and C4 (*P* < 0.001, **Figure 7H**). For RFS analysis, PCa patients with high riskscore + C2/C3 had poorer survival (*P* = 0.004, **Figures 7I, J**).

**Figure 8:**
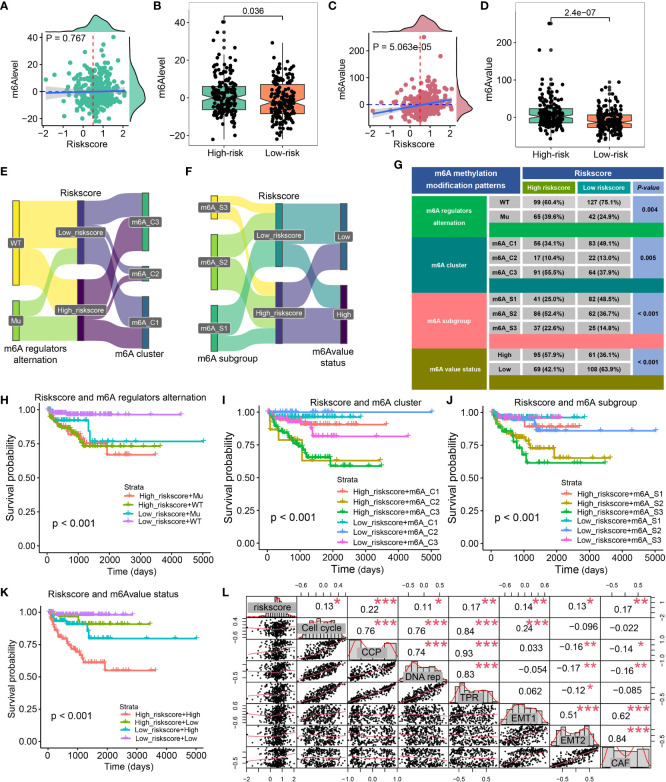
Association between CRPGS and m6A status. The association between riskscore and m6Alevel and m6Avalue **(A–D)**. Sanky plot showed the comparison results of CRPGS with m6A regulators alteration, m6A cluster, m6A subgroup, m6A value status **(E–F)**, and the χ^2^ test table showed the distribution of CRPGS in different m6A regulators alteration, m6A cluster, m6A subgroup, m6A value status groups **(G)**, and the combined effects of CRPGS + m6A regulators alteration/m6A cluster/m6A subgroup/m6A value status on PCa patients’ survival **(H–K)**. The correlation plot showed the association between CRPGS and other seven tumor signatures (Cell cycle, Cell cycle progression, DNA replication, Tumor Proliferation Rate, EMT1, EMT2, Cancer-associated fibroblasts) **(L)**. CAF, Cancer-associated fibroblasts; CCP, Cell cycle progression; DNA rep, DNA replication; EMT1, Epithelial-mesenchymal transition; TPR, Tumor Proliferation Rate. *p < 0.05; **p < 0.01; ***p < 0.001.

Zou et al (29) generate the m6Avalue and m6Alevel to assess the immune landscape, stemness, and drug response of PCa. High-risk patients exhibited a high m6Alevel (*P* = 0.036, **Figures 8A, B**), and riskscore was positively associative with m6Avalue, and patients with a high riskscore had a higher m6Avalue (all *P* < 0.001, **Figures 8C, D**). We also explore the distribution of riskscore in different molecular subtypes, including m6A regulators alternation, m6A cluster, m6A subgroup, and m6A value status, which was shown in **Figures 8E, F**. The contingency table found that most of the PCa patients belong to the m6A regulators wild type, while 39.9% of high-risk patients belong to the mutation type and only 24.9% of low-risk patients belong to the mutation type (*P* = 0.004, **Figure 8G**). Additionally, 55.5% of high-risk patients belong to the m6A cluster 3 while 49.1% of low-risk patients belong to the m6A cluster 1 (*P* = 0.005, **Figure 8G**), and 52.4% of high-risk patients belong to the m6A subgroup 2 while 48.5% of low-risk patients belong to m6A subgroup 1 (*P* < 0.001, **Figure 8G**), 57.9% of high-risk patients belong to high m6A value status while 63.9% of low-risk patients belong to low m6A value status (*P* < 0.001, **Figure 8G**). The CRPGS combined with the m6A regulators alternation, m6A cluster, m6A subgroup, and m6A value status could predict patients’ outcomes well (all *P* < 0.001, **Figures 8H–K**). We also found that CRPGS was positively associated with other seven tumor signatures, including Cell_cycle, Cell_cycle_progression (30), DNA_replication, Tumor_Proliferation_Rate (31), EMT1 (32), EMT2 (33), Cancer_associated_fibroblasts (31), indicating the potential role of the CRPGS in the progression and EMT of malignancy (**Figure 8L**).

### Construction of the CRPGS-based nomogram for RFS prediction in PCa

**Figure 9:**
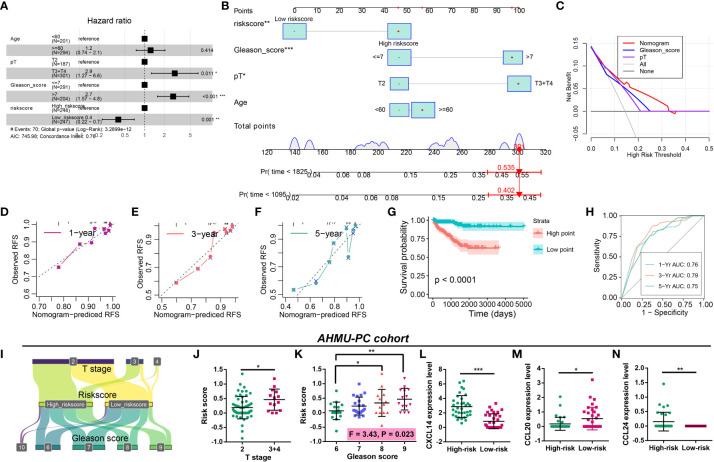
Construction of the CRPGS-based nomogram and validation of the CRPGS in the AHMU-PC cohort. Results of multivariate Cox regression with age, pathology T stage, Gleason score, and riskscore in PCa **(A)**. The CRPGS-based nomogram that integrated age, pathology T stage, Gleason score, and riskscore **(B)**, and the decision curve analysis and the 1/3/5-year calibration curves were used to evaluate the established nomogram **(C–F)**. Survival analysis between high- and low-nomogram-derived point **(G)**, and the point-based 1/3/5-year time-dependent ROC analysis were performed to assess the nomogram-derived point **(H)**. The Sanky plot showed the association between riskscore, T stage, and Gleason score **(I)**, and the distribution between patients with different T stages and Gleason scores **(J, K)**. The distribution in the expression levels of CXCL14, CCL20, and CCL24 in high- and low-risk patients **(L–N)**. *p < 0.05; **p < 0.01; ***p < 0.001.

The prognostic nomogram was constructed to better apply CRPGS into clinical practice. Multivariate Cox regression was performed, and pathology T stage, Gleason score, and riskscore were associated with RFS of PCa (**Figure 9A**). Age, pathology T stage, Gleason score, and riskscore were integrated into the prognostic nomogram, and the decision curve analysis showed the net benefit of the nomogram than Gleason and pathology T stage alone (**Figures 9B, C**), the 1/3/5-year calibration curves displayed the good agreement of the nomogram-predicted RFS and actual observed RFS (**Figures 9D–F**). In the CRPGS-based nomogram, low riskscore, Gleason score ≤ 7, pT2, and age < 60 were assigned with a point of 0, 47, 47, and 47, respectively, and high riskscore, Gleason > 7, pT3+T4, and age ≥ 60 were assigned with a point of 47, 97, 100, and 57, respectively. Based on the nomogram-derived point, we divided TCGA-PRAD patients into two groups, and patients with high points exhibited poorer survival, and the point-based 1/3/5-year time-dependent ROC analysis showed a good performance of the point, with an AUC of 0.76, 0.79, 0.75, respectively (**Figures 9G, H**).

### Validation of the CRPGS in real-world AHMU-PC cohort

The AHMU-PC cohort was used to validate the role of CRPGS in PCa. The Sanky plot showed the association among CRPGS, T stage (**Figure 9I**), and Gleason score, and patients with advanced T stage and Gleason score exhibited higher riskscore (**Figures 9J, K**). Consistent with the coefficients of the CRPGS, high-risk patients had higher CXCL14 and CCL24 expression, and lower CCL20 expression (**Figures 9L–N**).

## Discussion

For early-stage PCa, RP and radiotherapy were the recommended first-line options, and ADT-based therapy was the common therapy option for metastatic PCa (34, 35). For metastatic PCa, the 5-year survival was poor (36). Chemokines were involved in cell communication through three patterns (autocrine, endocrine, and paracrine). In PCa, chemokines were secreted by various cells (35), and chemokines played an important role in immune cell migration to regulate TME. CCL2 enhanced the angiogenesis, bone metastasis, and aggressiveness of PCa, blockade of CCL2 suppressed the growth of PCa (37). The expression of CXCL8 was positively associated with tumor stage, angiogenesis, and metastasis of PCa, and the elevated CXCL8 expression level was correlated with loss of AR expression (38, 39). In PCa, mesenchymal stem cells migrated into the tumor site through the chemotactic effects of CXCL16, which was further transformed into cancer-associated fibroblasts in TME, promoting the EMT and metastasis of PCa (40). Generally, biochemical recurrence refers to an elevated prostate-specific antigen (PSA) level post-RP, which was a predictor of disease progression (41). Hence, we explored the roles of chemokines in the BCR of PCa. We identified four BCR-related chemokine genes, including CXCL14, CCL20, CCL24, and CCL26, which were related to the progression and immune infiltration of PCa. Additionally, the identified four BCR-related chemokine genes were positively related to the ESTIMATE, immune, and stromal scores of PCa. Based on CXCL14, CCL20, CCL24, and CCL26, we defined PCa patients into two clusters, and compared to patients in cluster 1, cluster 2 patients exhibited poorer survival. The results of CIBERSORT showed patients in cluster1 and cluster 2 exhibited different cell levels infiltration, and cluster 2 patients had higher ESTIMATE, immune and stromal scores. Hence, CXCL14, CCL20, CCL24, and CCL26 were associated with the clinical and immune features of PCa, which further demonstrated their roles in regulating the TME of PCa (42).

Because CXCL14, CCL20, CCL24, and CCL26 exerted significant roles in PCa, the CRPGS was established based on the four identified chemokine genes, and PCa patients with high riskscores exhibited poorer survival than patients with low riskscores. Patients with high riskscores exhibited advanced pathology stages. Moreover, riskscore was positively associated with ESTIMATE and stromal scores, and patients in low- and high-risk groups had different immune cell infiltration levels. We further explored the association between riskscore, IPS, and immune checkpoint to investigate the effects of CRPGS on PCa immunotherapy. High-risk patients had lower IPS, IPS-PD1, and IPS-CTLA4 scores, and high-risk patients also had lower levels of CD40, CEACAM1, LGALS3, and TNFRSF14. IPS consists of four categories, including effector and immunosuppressive cells, immunomodulators, and MHC molecules (18). IPS was positively associated with tumor immunogenicity, and IPS was used to predict patients’ responses to immunotherapy. Therefore, we proposed that PCa low-risk patients had higher immunogenicity and may respond better to immunotherapy. Consistently, the results of TIDE further demonstrated that low-risk patients had lower TIDE scores, indicating that low-risk patients respond better to immunotherapy. Furthermore, a higher proportion of low-risk patients were sensitive to immunotherapy in the GSE78220 cohort. Recently, m6A was used to predict patients’ responses to immunotherapy. Zou et al. proposed that PCa patients with low m6Avalues respond better to immunotherapy and had a longer survival time (29). Consistently, our results indicated that the CRPGS was positively associated with m6Avalue, and low-risk PCa patients had lower m6Avalues and m6Alevels, which implied that low-risk patients exhibited lower levels of m6A status and had a better response to immunotherapy. Hence, low-risk patients possessed higher immunogenicity and benefited more from immunotherapy.

To explore the different pathway activities between low- and high-risk patients, we performed GSVA between these two groups. We found that the androgen response pathway activity was decreased in high-risk patients, which indicated that PCa patients with different riskscores may have different sensitivity to anti-androgen therapy. Yu et al. identified several androgen-responsive elements in the promoter of CXCR4 and CXCR7, and the CXCL12/CXCR4 and CXCL12/CXCR7 axes may be regulated by the AR signaling pathway and TLR5 ligand (flagellin) in PCa (43), implying the potential relationship between chemokines and ADT response. Therefore, we explored the association between CRPGS and ADT response, and we found that the CCL20 expression level was increased after ADT. Then, we performed GSVA to quantify the AR signaling activity. Consistently, our results showed that PCa patients with lower riskscores had higher AR activity, indicating a better response to ADT. PCa patients with low risksocre exhibited lower estimated IC50 of bicalutamide, further indicating that low-risk patients may respond better to ADT.

Currently, a growing number of prognostic molecular signatures were established to classify patients into different groups based on the underlying molecular features. In our study, We compared CRPGS with our previously established molecular signatures. We previously classified the TCGA-PRAD patients into non-immune, immune-activated, and immune-suppressed subtypes (16), and patients in the immune-activated group responded better to ICB therapy, Additionally, the PCa multi-omics classification (PMOC) system based on multi-omic data were also established in our previous study (24), and PCa patients in PMOC3 subtype respond better to ADT. By comparing CRPGS with the two previously established PCa-related molecular signatures (16, 24), we found that most PCa patients were in the non-immune group, and a higher proportion of high-risk patients belong to the immune-suppressed group, which had poorer survival. Additionally, most PCa patients belong to the PMOC1 subtypes, 24.6% of high-risk and 38.8% of low-risk patients belong to the PMOC3 subtype, respectively, indicating low-risk patients may respond better to ADT.

Additionally, we compared the CRPGS with another three molecular subtypes. Zhao et al (26) developed a PAM50 classifier and divided the 22 carcinomas into luminal A, luminal B, and basal-like subtypes. More than half of high-risk patients possessed luminal B subtypes, and patients with high-risk + luminal B subtype had poor RFS, which was in agreement with Zhao et al’ results that patients with luminal B subtype had the poorest outcomes (44). Thorsson et al (28) developed another immune signature across 33 cancer types, which constituted six subtypes, and four subtypes were identified in PCa, including inflammatory, wound healing, IFN-γ dominant, lymphocyte Depleted, and we found that most PCa patients belong to the inflammatory subtypes. Tamborero et al (27) developed six immunophenotypes across 29 cancer types, including lowly (1 and 2 subtypes), intermediately (3 and 4 subtypes), and highly (5 and 6 subtypes) cytotoxic immunophenotypes, and they found that patients with high cytotoxic immunophenotypes exhibited better prognosis. Compared with Tamborero et al’s study (27), we found no difference in the distribution of PCa patients between CRPGS and the immunophenotype, while CRPGS + immunophenotype may be used to predict patients’ survival.

The Cancer Genome Atlas Research Network allocated PCa patients into seven molecular subtypes (25), including fusions in ERG, ETV1, ETV4, FLI1, and mutations in SPOP, FOXA1, IDH1. Based on the study (25), we found that PCa patients with TP53 and SPOP mutation had higher riskscores, and patients with SETD2 mutation had lower riskscores compared to wild ones. In PCa, SPOP was identified as a tumor suppressor, and SPOP was associated with the ERG protein stability (45) and the growth and aggressiveness of PCa (46, 47). Therefore, SPOP mutation may lead to poor outcomes for PCa patients, which was similar to our results that high-risk patients with higher SPOP mutation, and exhibited poor survival. TP53 mutation was also associated with the increased aggressiveness and poor prognosis of PCa, and the TP53 mutation-based mutation signature could predict patients’ outcomes (48), which was consistent with our results that PCa patients with TP53 mutation had higher riskscores, indicating poor prognosis. CRPGS was also positively related to the Cell cycle, Cell cycle progression (30), DNA replication, Tumor Proliferation Rate, EMT1, EMT2, and Cancer-associated fibroblasts signatures, which further demonstrated the important role of CRPGS in the progression of PCa.

Because the CRPGS had significant roles in the progression and drug therapy response in PCa, we established the CRPGS-based nomogram to apply CRPGS into practice, which exhibited good performance in RFS prediction. Moreover, we validated the CRPGS in our real-world AHMU-PC cohort, and the results were consistent with our bioinformatic results, indicating the wide application of CRPGS.

There are some limitations to our study. The four chemokine genes (CXCL14, CCL20, CCL24, and CCL26) were identified by the LASSO-Cox regression, and their roles were predicted by bioinformatics analysis and validated in the AHMU-PC cohort, and the experimental study was not performed to further assess their effects on PCa. Additionally, TCGA and GEO datasets were used in our study, and the heterogenicity between different cohorts should not be ignored. The role of CRPGS was validated in the AHMU-PC cohort, while the mutation data of SPOP and TP53 was extracted from the public dataset, which was not validated in our AHMU-PC cohort. Although the association between CRPGS and ADT and immunotherapy responses were analyzed by different methods, including TIDE, CellMiner, CARE, and GEO cohorts, the ADT and immunotherapy data was not obtained in our AHMU-PC cohort, thus, the effects of CRPGS on ADT and immunotherapy were not validated in AHMU-PC cohort. Besides, not all low-risk PCa patients benefited from anti-androgen and immunotherapies in our study, therefore, the CRPGS was not the only factor related to anti-androgen and immunotherapies responses. In the future, we would explore the factors related to the therapy efficacies of ADT and immunotherapy to achieve the maximum effects of drugs therapy in PCa.

## Conclusions

The chemokine genes played an important role in the progression of PCa, and the established CRPGS was associated with the clinical, pathological, and immune characteristics of PCa. The CRPGS could be used to predict patients’ responses to ADT and immunotherapy, and the CRPGS-based nomogram performed well in RFS prediction.

## Data availability statement

The original contributions presented in the study are included in the article/**Supplementary Material**, further inquiries can be directed to the corresponding authors.

## Ethics statement

The studies involving human participants were reviewed and approved by ethical committee of the First Affiliated Hospital of Anhui Medical University. Written informed consent for participation was not required for this study in accordance with the national legislation and the institutional requirements.

## Author contributions

LC, YZ, and CJ analyzed data, drew illustrations, and wrote the manuscript; CY, LZ and CL designed the study and revised the manuscript. All authors contributed to this manuscript, and all authors read and approved the final manuscript.

## Funding

This work was supported by the Research Fund of Anhui Institute of Translational Medicine (No. ZHYX2020A003), Supporting Project for Distinguished Young Scholar of Anhui Colleges. National Natural Science Foundation of China (82270818, 81700662).

## Acknowledgments

We thank TCGA, GEO projects, and the data cohorts used in our study for providing the valuable datasets.

## Conflict of interest

The authors declare that the research was conducted in the absence of any commercial or financial relationships that could be construed as a potential conflict of interest.

## Publisher’s note

All claims expressed in this article are solely those of the authors and do not necessarily represent those of their affiliated organizations, or those of the publisher, the editors and the reviewers. Any product that may be evaluated in this article, or claim that may be made by its manufacturer, is not guaranteed or endorsed by the publisher.

## Abbreviations

ADT, androgen deprivation therapy; AHMU-PC, Anhui Medical University-Prostate Cancer; AR, androgen receptor; ARA, AR activity; ARSI, androgen receptor signaling inhibitor; BCR, biochemical recurrence; CARE, Computational Analysis of Resistance; CR, complete response; CRPGS, chemokine-related prognostic gene signature; GSE, gene set enrichment; GSEA, Gene Set Enrichment Analysis; IPS, immunophenoscore; MCODE, Molecular COmplex Detection; PCa, Prostate cancer; PD, progressive disease; PMOC, PCa multi-omics classification; PLNR, positive lymph node ratio; PR, partial response; PSA, prostate-specific antigen; RFS, recurrence-free survival; RP, radical prostatectomy; SD, stable disease; TCGA-PRAD, The Cancer Genome Atlas-Prostate Adenocarcinoma; TME, tumor microenvironment.
