# Supplementary material for: The established chemokine-related prognostic gene signature in prostate cancer: Implications for anti-androgen and immunotherapies
Source: Front Immunol. 2022 Oct 6;13:1009634. doi: 10.3389/fimmu.2022.1009634 (PMC9582844; doi:10.3389/fimmu.2022.1009634)
Supplement: Supplementary file 1 [file DataSheet_1.docx]

**The Established Chemokine-Related Prognostic Gene Signature in Prostate Cancer: Implications for Anti-androgen and Immunotherapies**

Lei Chen^1,2,3^, Yi Zheng^1,2,3^, Changqin Jiang^1,2,3^, Cheng Yang^1,2,3^*, Li Zhang^1,2,3^*, Chaozhao Liang^1,2,3^*

^1^Department of Urology, The First Affiliated Hospital of Anhui Medical University, Hefei, China; ^2^Institute of Urology, Anhui Medical University, Hefei, China;

^3^Anhui Province Key Laboratory of Genitourinary Diseases, Anhui Medical University, Hefei, China.

**Correspondence to** Cheng Yang ([chengyang_md@163.com](mailto:chengyang_md@163.com)), Li Zhang ([lzhang@ahmu.edu.cn](file:///E:\科研\Cancer%20Medicine_Revision_Submission\lzhang@ahmu.edu.cn)) & Chaozhao Liang ([liang_chaozhao@ahmu.edu.cn](mailto:liang_chaozhao@ahmu.edu.cn)).

**Tel.:** +86 19955195611, and Fax.: +86 19955195611

**Address:** Jixi Road 218, Shushan District, Hefei City 230022, Anhui Province, People’s Republic of China.

**Running title:** The chemokine-related gene signature in PCa.

**Supplemental Materials and Methods**

**Establishment of chemokine-related prognostic gene signature (CRPGS) in PCa**

The gene expression levels of 57 chemokine genes (C-X-C and C-C motif) were extracted from the TCGA-PRAD cohort in the form of log2(TPM+1). The biological function of the 57 chemokine genes was illustrated by the Metascape online tool (<https://metascape.org/>). We performed univariate Cox regression to select RFS-related chemokine genes, and the LASSO-Cox regression further identified RFS-related chemokine genes and established the CRPGS in the TCGA-PRAD cohort. The association between identified genes and PCa patients’ clinical characteristics was analyzed. We calculated the immune, stromal, and ESTIMATE scores of PCa patients by the ESTIMATE algorithm[1], and the association between the identified genes and immune, stromal, and ESTIMATE scores was evaluated. The TIMER online tool (<https://cistrome.shinyapps.io/timer/>) was used to explore the association between the identified genes and tumor immune infiltration level[2, 3]. The distribution of the identified genes in different single-cell types of the prostate was determined by the Human Protein Atlas project (HPA, <https://www.proteinatlas.org/>). The CRPGS was as follow: Riskscore = α1 * Exp1 + α2 * Exp2 +…+ αi * Expi. The α represented the coefficient of the identified gene in the LASSO-Cox regression analysis, and Exp represented the expression level of the identified genes. The ROC, time-dependent ROC, and time-dependent AUC analyses were performed to evaluate the sensitivity and specificity of CRPGS with “survival”, “ROCR”, and “timeROC” R packages. Based on the median value of CRPGS riskscore, we classified TCGA-PRAD patients into low- and high-risk groups, and survival analyses were performed between these two subgroups by using the “survminer” R package. The CRPGS was validated in the GSE70768 cohort.

**Gene set variation analysis (GSVA)**

GSVA belongs to the unsupervised and non-parametric gene set enrichment (GSE) method to obtain the GSE score and reflect the variation of pathway activity in a certain population[4]. We downloaded the 50 hallmark gene sets from the Gene Set Enrichment Analysis (GSEA) database (<https://www.gsea-msigdb.org/gsea/index.jsp>) [5]. We performed GSVA between low- and high-risk PCa patients in five PCa cohorts (GSE70768, GSE70769, GSE46602, and TCGA-PRAD), and the activated or suppressed pathways were extracted and the Venn diagram was used to identify the commonly activated of suppressed pathways from the GSVA results of the four cohorts using the “GSVA” package[4].

**Consensus cluster analysis**

Based on the identified four chemokine genes in TCGA-PRAD cohort, PCa patients were clustered using the “ConsensusClusterPlus” R package with the following parameters: 50 repeats, *k*_max_ = 10, and agglomerative hierarchical clustering with Pearson distance. Based on the consistency within the cluster, variation in coefficient, and the increase in the area under the consensus cumulative distribution function (CDF) curve, the optimal number of clusters was selected. Survival analysis was performed between different clusters, and the distribution of immune cells between different clusters was evaluated by the CIBERSORT method [6]. The difference in immune, stromal, and ESTIMATE scores between different clusters was quantified by the ESTIMATE algorithm[1].

**The association between CRPGS and PCa patients’ clinical and immune characteristics**

The expression levels of four identified chemokine genes between low- and high-risk subgroups were compared. The distribution of riskscores in PCa patients with different BCR statuses, Gleason scores, pathology T and N stages, clinical T stages, positive lymph node ratio (PLNR), and residual tumor statuses were analyzed. PLNR refers to the ratio of positive lymph node number to total examined lymph node number, and we regarded the 10% of PLNR as the cut-off value. The association between riskscore and immune, stromal, and ESTIMATE scores was explored, and the distribution of 22 types of immune in high- and low-risk subgroups was analyzed to investigate the immunological alteration between these two subgroups. Based on The Cancer Immunome Atlas (TCIA) database[7], TCGA-PRAD patients’ immunophenoscore (IPS) was calculated, which was used to evaluate the response to immunotherapies. TCGA-PRAD patients’ CYT scores were quantified to estimate the immune cytolytic activities of PCa [8]. The distribution of IPS and CYT scores between low- and high-risk subgroups was compared.

**The identified chemokine genes and drug response**

The Computational Analysis of REsistance (CARE, <http://care.dfci.harvard.edu/>) is an online tool to predict the association between genes and the targeted drug efficacy based on Genomics of Drug Sensitivity in Cancer (GDSC, also known as CGP), Cancer Cell Line Encyclopedia (CCLE), and the Cancer Therapeutics Response Portal (CTRP) datasets[9]. In our study, we explored the association between the identified chemokine genes and drug sensitivity from the GDSC, CCLE, and CTRP databases. The CellMiner (<https://discover.nci.nih.gov/cellminer/>) was used to analyze the relationship between drug efficacy and gene expression based on the NCI-60 cell and other cancer cells[10]. We downloaded the relative data files from the CellMiner, and analyzed the association between the identified chemokine gene expression levels and drug response using R software with “impute”, “limma”, “ggplot2”, and “ggpubr” packages.

**The association between CRPGS, androgen deprivation therapy (ADT), and immunotherapy**

Based on the results from GSVA, we further explored the association between CRPGS and ADT response. Bicalutamide, a nonsteroidal AR antagonist, was recommended for advanced PCa[11]. We calculated the estimated IC50 of bicalutamide between low- and high-risk patients using the “pRRophetic” package [12, 13] in TCGA-PRAD and Abida *et al*’s cohort [14]. The alterations in the expression levels of CXCL14, CCL20, CCL24, and CCL26 were estimated in PCa patients before and after ADT therapy in the GSE150368 cohort. In Abida *et al*’s cohort[14], we extracted the gene expression matrix and AR signaling inhibitor (ARSI) treatment status, and the AR activity (ARA) score was calculated to quantify AR signaling activity by “GSVA” package based on nine AR signaling genes (KLK3, ACSL3, FKBP5, KLK2, PPAP2A, STEAP1, STEAP2, RAB3B, NKX3-1)[15]. The association between CRPGS and ARSI was analyzed in Abida *et al*’s cohort.

To explore the association between riskscore and immunotherapy, the difference in immune checkpoint levels between different risk groups was analyzed. Additionally, the GSE78220 cohort and TIDE score were also used. The GSE78220 contained 27 melanoma patients receiving anti-PD-1 therapy, and the riskscore of these 27 melanoma patients was calculated based on the coefficients of CXCL14, CCL20, CCL24, and CCL26 and the corresponding expression levels, and the association between CRPGS and patients’ therapy response were analyzed. Moreover, the TIDE score of each sample in the TCGA-PRAD and GSE70768 cohorts was calculated by the online tool (<http://tide.dfci.harvard.edu/>) [16, 17].

**Comparison of CRPGS with other molecular signatures**

Sankey plot was used to visualize the distribution of TCGA-PRAD patients in our CRPGS and other published molecular signature, and the χ^2^ test was used to examine the distribution difference between the CRPGS and other signatures. Firstly, we compared the CRPGS with our two previously established two PCa-related molecular signatures[18, 19]. We previously classified the TCGA-PRAD patients into non-immune, immune-activated, and immune-suppressed subtypes by the non-negative matrix factorization and the nearest template prediction methods, and further validated our novel immune molecular classifier in other GEO-PCa and our Anhui Medical University-Prostate Cancer (AHMU-PC) cohorts[18], and PCa patients with the immune-activated status benefited more from immunotherapy, which could assist the clinician to assess patients’ response to immunotherapy. Additionally, we also developed the PCa multi-omics classification (PMOC) system based on multi-omic data, including mRNA, long non-coding RNA, microRNA, somatic mutation, and DNA methylation[19]. We found that patients in the PMOC3 subtype may have a better response to ADT treatment, and the PMOC system may promote the treatment decision on PCa.

Next, the CRPGS was also compared with another three published molecular subtypes. Zhao *et al*[20] performed a pan-cancer analysis and developed a PAM50 classifier to divide the 22 carcinomas into luminal (luminal A and luminal B) and basal-like subtypes. Thorsson *et al*[21] defined six immune subtypes across 33 cancer types, including inflammatory, wound healing, IFN-γ dominant, lymphocyte Depleted, TGF-β Dominant, and immunologically quiet. Tamborero *et al*[22] identified six immunophenotypes across 29 solid cancers, which were further subtyped as lowly (1 and 2 subtypes), intermediately (3 and 4 subtypes), highly (5 and 6 subtypes) cytotoxic immunophenotypes, and patients with high cytotoxic immunophenotypes tend to have better survival. The Cancer Genome Atlas Research Network classified 76% of 333 primary TCGA-PCa into seven molecular subtypes[23], including fusions in ERG, ETV1, ETV4, FLI1, and mutations in SPOP, FOXA1, IDH1, and the remained 24% was characterized as other subtypes. The association between riskscore and SPOP, TP53, SETD2 mutations, and ERG fusion was analyzed. The Cancer Genome Atlas Research Network also performed DNA methylation cluster analysis, which divided TCGA-PRAD samples into C1, C2, C3, and C4 clusters, and the association between riskscore and methylation cluster was analyzed. Finally, the combined effect of riskscore + molecular classifier on RFS of PCa patients was performed.

Zou *et al*[24] generated the m6Avalue and m6Alevel to assess the immune landscape, stemness, and drug response of PCa, and they also classified the TCGA-PRAD sample into different groups based on the m6A regulators alternation, m6A cluster, m6A subgroup, m6A value status. We explored the distribution of riskscore in different molecular subtypes, including m6A regulators alteration, m6A cluster, m6A subgroup, and m6A value status. Survival analysis was used to explore the combined effects of riskscore and molecular signatures on PCa patients’ prognosis. We also investigated the association between riskscore and other seven tumor signatures, including Cell_cycle (<https://www.kegg.jp/pathway/hsa04110>), Cell_cycle_progression[25], DNA_replication (<https://www.kegg.jp/pathway/map03030>), Tumor_Proliferation_Rate[26], EMT1[27], EMT2[28], and Cancer_associated_fibroblasts[26], to explore the potential role of CRPGS in the progression and EMT of PCa.

**Establishment of the CRPGS-based nomogram for RFS prediction**

Because tumor stage, Gleason score, and age were closely related to the prognosis of PCa, we performed multivariate Cox regression by integrating pT stage, Gleason score, age, and riskscore. The CRPGS-based nomogram was established by integrating pT, Gleason score, age, and riskscore, and the decision curve and 1-/3-/5-year calibration curve analyses were used to evaluate the net benefit and predictivity of the established nomogram. Based on the established nomogram, each patient in the TCGA-PRAD cohort obtained a nomogram point, and we divided patients into low- and high-point groups based on the point median value, and survival and time-dependent ROC analyses were performed to evaluate the predictivity of the nomogram point, which was performed in R software with “rms”, “survival”, “regplot”, and “rmda” packages.

**Reference**

1 Yoshihara K, Shahmoradgoli M, Martínez E, Vegesna R, Kim H, Torres-Garcia W *et al*. Inferring tumour purity and stromal and immune cell admixture from expression data. *Nat Commun* 2013; 4: 2612.

2 Li T, Fan J, Wang B, Traugh N, Chen Q, Liu JS *et al*. TIMER: A Web Server for Comprehensive Analysis of Tumor-Infiltrating Immune Cells. *Cancer Res* 2017; 77: e108-e110.

3 Li T, Fu J, Zeng Z, Cohen D, Li J, Chen Q *et al*. TIMER2.0 for analysis of tumor-infiltrating immune cells. *Nucleic Acids Res* 2020; 48: W509-w514.

4 Hänzelmann S, Castelo R, Guinney J. GSVA: gene set variation analysis for microarray and RNA-seq data. *BMC Bioinformatics* 2013; 14: 7.

5 Subramanian A, Tamayo P, Mootha VK, Mukherjee S, Ebert BL, Gillette MA *et al*. Gene set enrichment analysis: a knowledge-based approach for interpreting genome-wide expression profiles. *Proc Natl Acad Sci U S A* 2005; 102: 15545-15550.

6 Newman AM, Liu CL, Green MR, Gentles AJ, Feng W, Xu Y *et al*. Robust enumeration of cell subsets from tissue expression profiles. *Nat Methods* 2015; 12: 453-457.

7 Charoentong P, Finotello F, Angelova M, Mayer C, Efremova M, Rieder D *et al*. Pan-cancer Immunogenomic Analyses Reveal Genotype-Immunophenotype Relationships and Predictors of Response to Checkpoint Blockade. *Cell Rep* 2017; 18: 248-262.

8 Rooney MS, Shukla SA, Wu CJ, Getz G, Hacohen N. Molecular and genetic properties of tumors associated with local immune cytolytic activity. *Cell* 2015; 160: 48-61.

9 Jiang P, Lee W, Li X, Johnson C, Liu JS, Brown M *et al*. Genome-Scale Signatures of Gene Interaction from Compound Screens Predict Clinical Efficacy of Targeted Cancer Therapies. *Cell Syst* 2018; 6: 343-354.e345.

10 Reinhold WC, Sunshine M, Liu H, Varma S, Kohn KW, Morris J *et al*. CellMiner: a web-based suite of genomic and pharmacologic tools to explore transcript and drug patterns in the NCI-60 cell line set. *Cancer Res* 2012; 72: 3499-3511.

11 Vaishampayan UN, Heilbrun LK, Monk P, 3rd, Tejwani S, Sonpavde G, Hwang C *et al*. Clinical Efficacy of Enzalutamide vs Bicalutamide Combined With Androgen Deprivation Therapy in Men With Metastatic Hormone-Sensitive Prostate Cancer: A Randomized Clinical Trial. *JAMA Netw Open* 2021; 4: e2034633.

12 Geeleher P, Cox NJ, Huang RS. Clinical drug response can be predicted using baseline gene expression levels and in vitro drug sensitivity in cell lines. *Genome Biol* 2014; 15: R47.

13 Geeleher P, Cox N, Huang RS. pRRophetic: an R package for prediction of clinical chemotherapeutic response from tumor gene expression levels. *PLoS One* 2014; 9: e107468.

14 Abida W, Cyrta J, Heller G, Prandi D, Armenia J, Coleman I *et al*. Genomic correlates of clinical outcome in advanced prostate cancer. *Proc Natl Acad Sci U S A* 2019; 116: 11428-11436.

15 Faisal FA, Sundi D, Tosoian JJ, Choeurng V, Alshalalfa M, Ross AE *et al*. Racial Variations in Prostate Cancer Molecular Subtypes and Androgen Receptor Signaling Reflect Anatomic Tumor Location. *Eur Urol* 2016; 70: 14-17.

16 Fu J, Li K, Zhang W, Wan C, Zhang J, Jiang P *et al*. Large-scale public data reuse to model immunotherapy response and resistance. *Genome Med* 2020; 12: 21.

17 Jiang P, Gu S, Pan D, Fu J, Sahu A, Hu X *et al*. Signatures of T cell dysfunction and exclusion predict cancer immunotherapy response. *Nat Med* 2018; 24: 1550-1558.

18 Meng J, Zhou Y, Lu X, Bian Z, Chen Y, Zhou J *et al*. Immune response drives outcomes in prostate cancer: implications for immunotherapy. *Mol Oncol* 2021; 15: 1358-1375.

19 Meng J, Lu X, Jin C, Zhou Y, Ge Q, Zhou J *et al*. Integrated multi-omics data reveals the molecular subtypes and guides the androgen receptor signalling inhibitor treatment of prostate cancer. *Clin Transl Med* 2021; 11: e655.

20 Zhao SG, Chen WS, Das R, Chang SL, Tomlins SA, Chou J *et al*. Clinical and Genomic Implications of Luminal and Basal Subtypes Across Carcinomas. *Clin Cancer Res* 2019; 25: 2450-2457.

21 Thorsson V, Gibbs DL, Brown SD, Wolf D, Bortone DS, Ou Yang TH *et al*. The Immune Landscape of Cancer. *Immunity* 2018; 48: 812-830.e814.

22 Tamborero D, Rubio-Perez C, Muiños F, Sabarinathan R, Piulats JM, Muntasell A *et al*. A Pan-cancer Landscape of Interactions between Solid Tumors and Infiltrating Immune Cell Populations. *Clin Cancer Res* 2018; 24: 3717-3728.

23 The Molecular Taxonomy of Primary Prostate Cancer. *Cell* 2015; 163: 1011-1025.

24 Zou C, He Q, Feng Y, Chen M, Zhang D. A m(6)Avalue predictive of prostate cancer stemness, tumor immune landscape and immunotherapy response. *NAR Cancer* 2022; 4: zcac010.

25 Cuzick J, Berney DM, Fisher G, Mesher D, Møller H, Reid JE *et al*. Prognostic value of a cell cycle progression signature for prostate cancer death in a conservatively managed needle biopsy cohort. *Br J Cancer* 2012; 106: 1095-1099.

26 Bagaev A, Kotlov N, Nomie K, Svekolkin V, Gafurov A, Isaeva O *et al*. Conserved pan-cancer microenvironment subtypes predict response to immunotherapy. *Cancer Cell* 2021; 39: 845-865.e847.

27 Damrauer JS, Hoadley KA, Chism DD, Fan C, Tiganelli CJ, Wobker SE *et al*. Intrinsic subtypes of high-grade bladder cancer reflect the hallmarks of breast cancer biology. *Proc Natl Acad Sci U S A* 2014; 111: 3110-3115.

28 Hugo W, Zaretsky JM, Sun L, Song C, Moreno BH, Hu-Lieskovan S *et al*. Genomic and Transcriptomic Features of Response to Anti-PD-1 Therapy in Metastatic Melanoma. *Cell* 2016; 165: 35-44.

**Supplemental Figures and Legends**

**
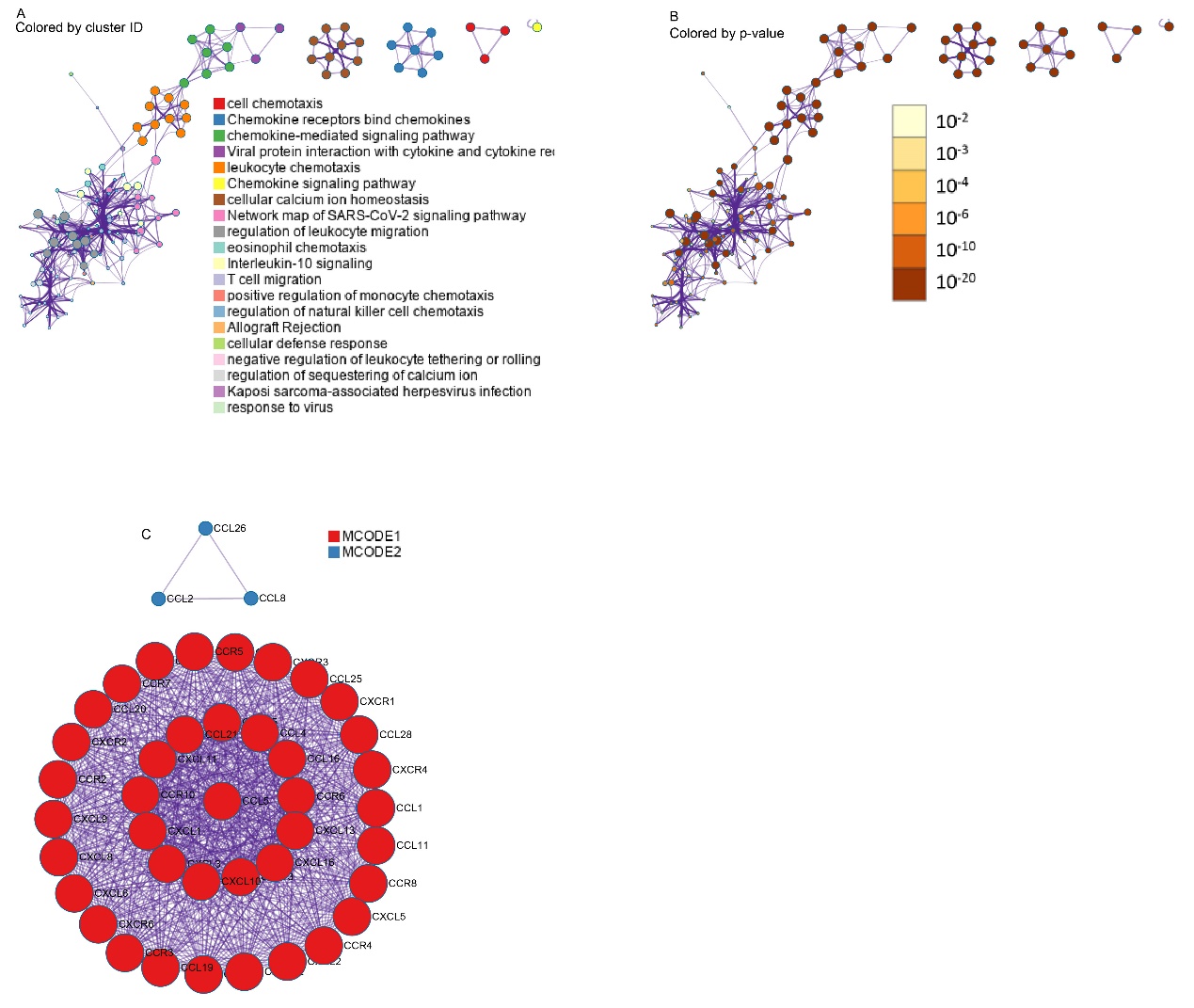
Supplemental Figure 1. Functional annotation of chemokine genes.** The enrichment analysis of the 57 chemokine genes (**A-B**), and the 57 chemokine genes were divided into two models by Molecular COmplex Detection (MCODE) (**C**).

**
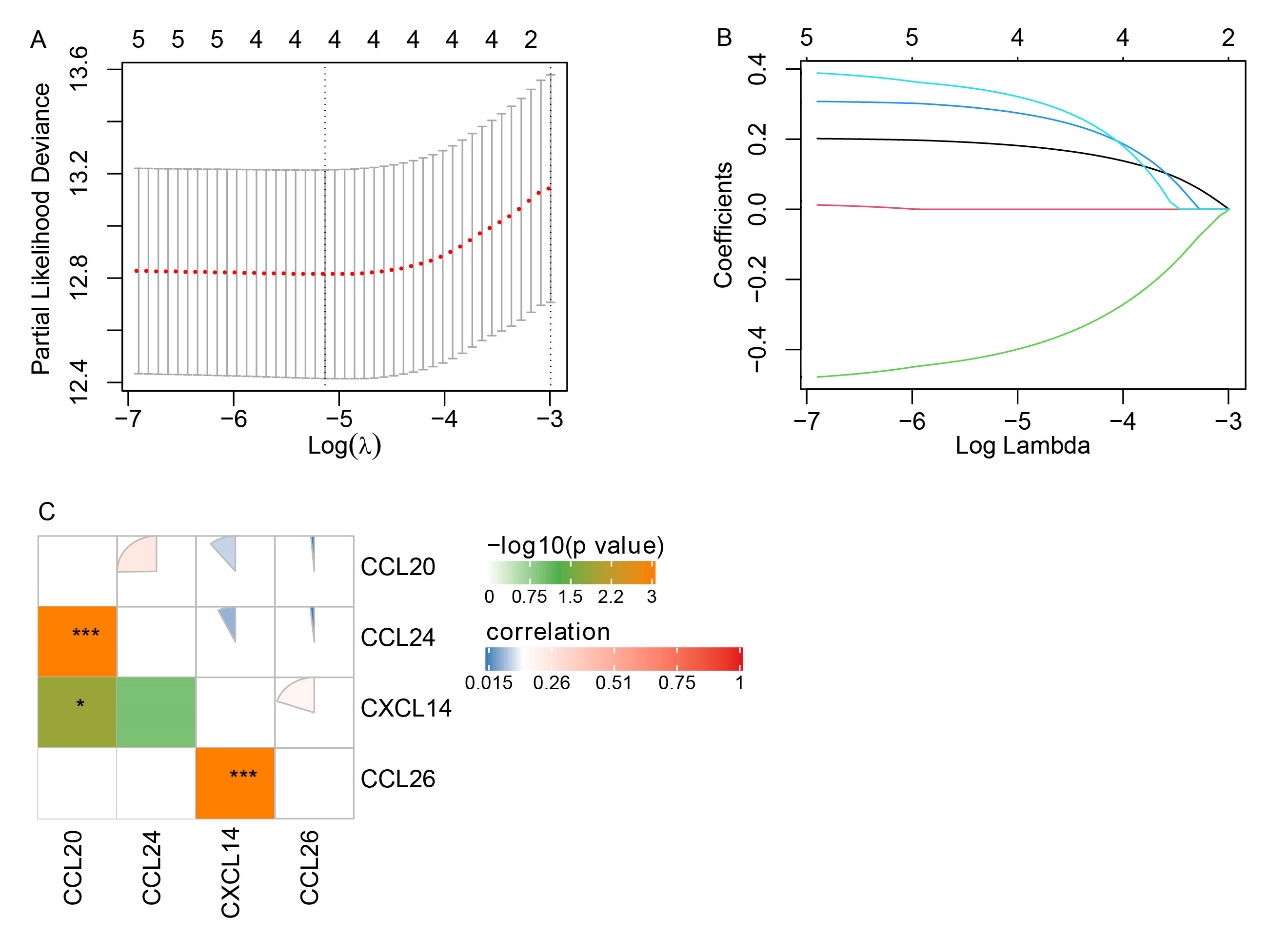
Supplemental Figure 2. LASSO-Cox results that based on the 57 chemokine genes.** Results of the LASSO-Cox regression identified the BCR-related chemokine genes (**A-B**), and the association among these four chemokine genes was analyzed (**C**).


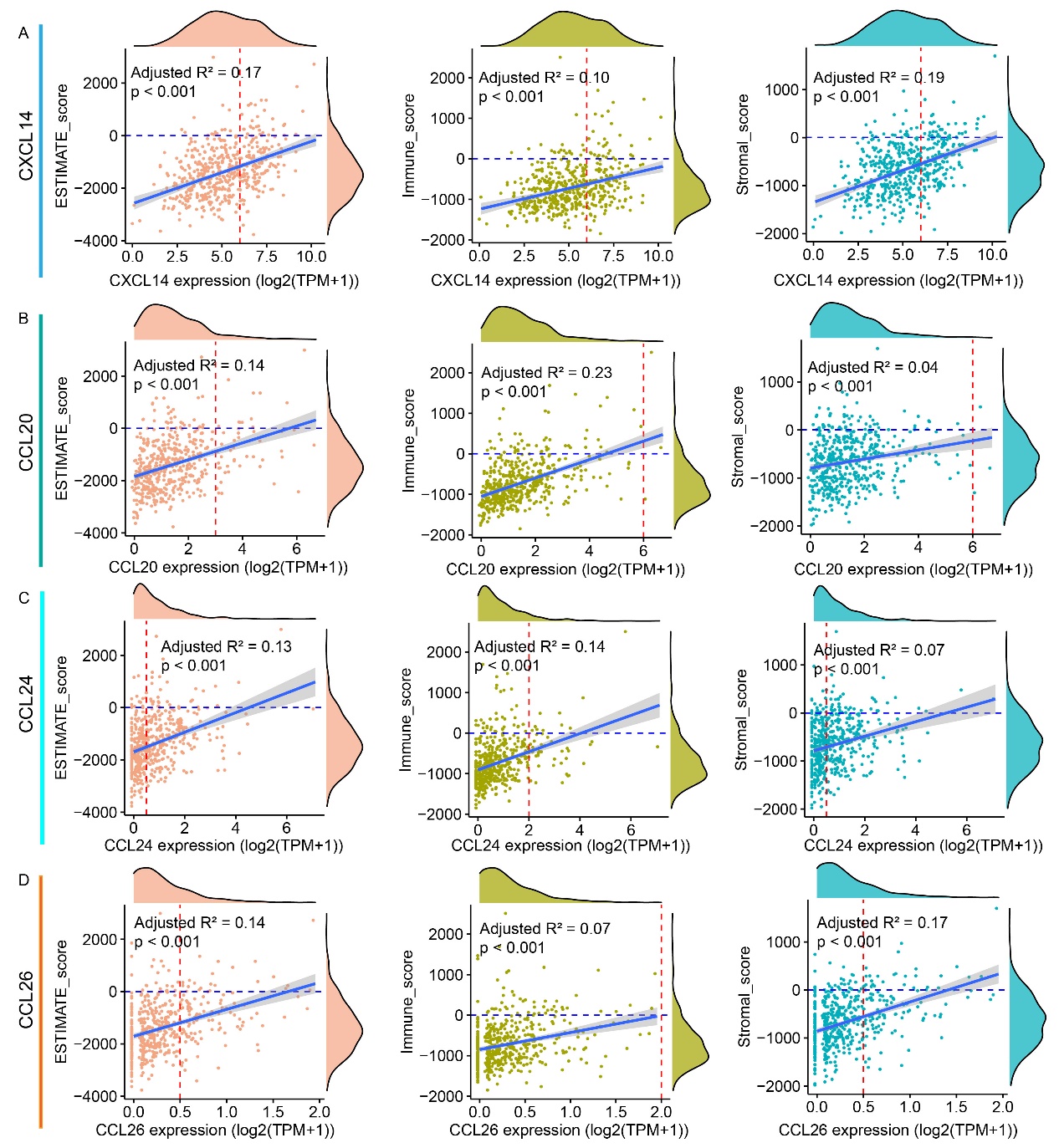


**Supplemental Figure 3. The association between CXCL14, CCL20, CCL24, CCL26, and ESTIMATE score, immune score, and stromal scores of PCa (A-D).**


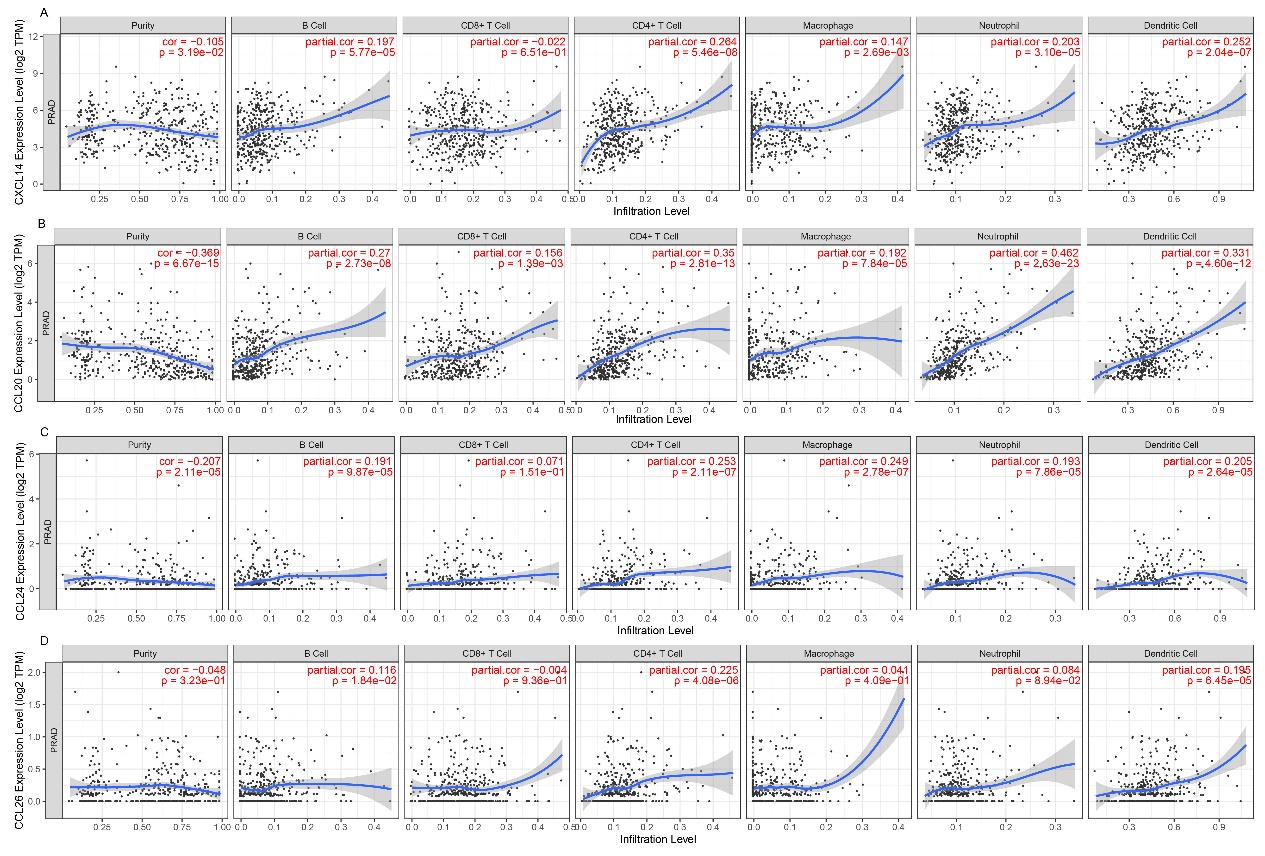


**Supplemental Figure 4. The association between CXCL14, CCL20, CCL24, CCL26, and tumor purity and immune infiltration of PCa (A-D).**

**
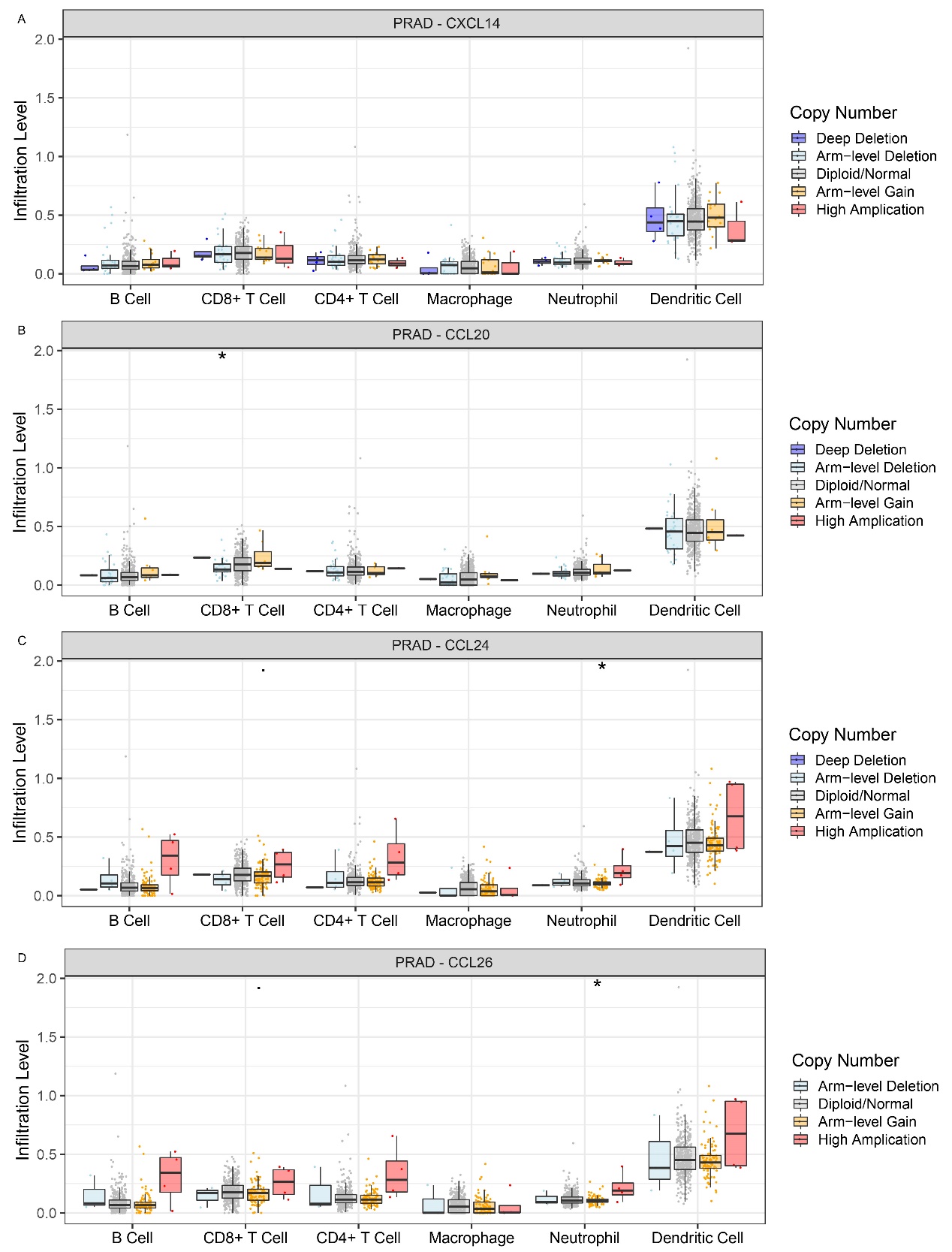
**

**Supplemental Figure 5. The association between somatic copy number alterations of CCL20, CCL24, and CCL26 ad immune infiltration of PCa (A-D).**

**
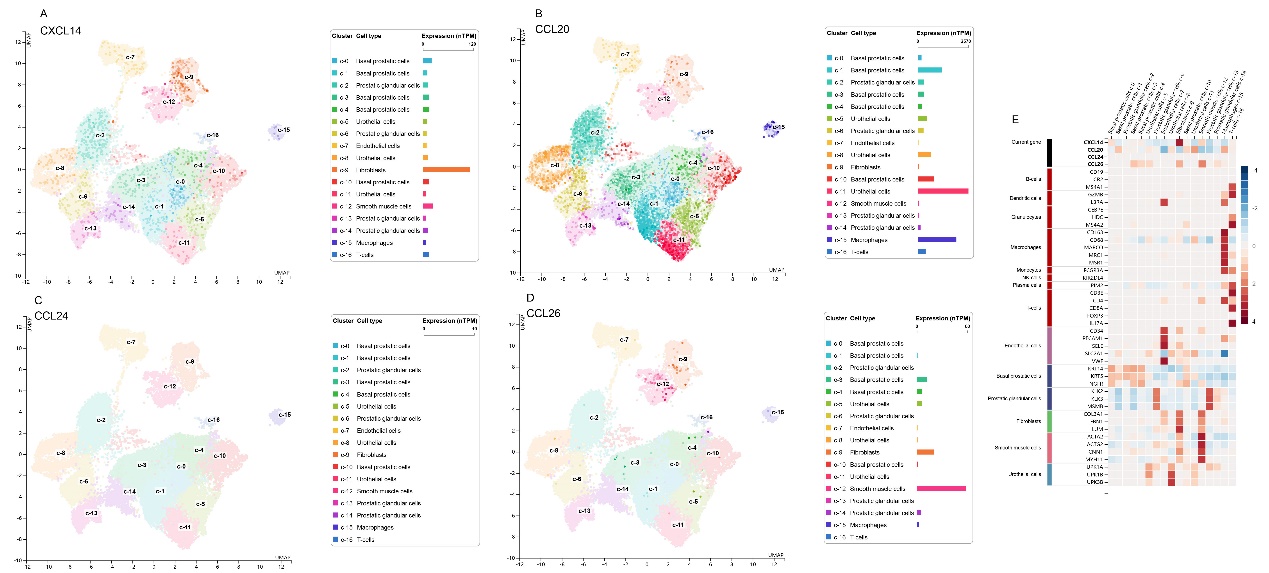
Supplemental Figure 6. Distribution of CXCL14, CCL20, CCL24, and CCL26 in different cell types by the single-cell RNA sequencing analysis.** Single-cell RNA sequence of human prostate tissue showed the expression of CXCL14, CCL20, CCL24, and CCL26 in different cell types (**A-D**), and the association between CXCL14, CCL20, CCL24, and CCL26 and various cell markers (**E**).


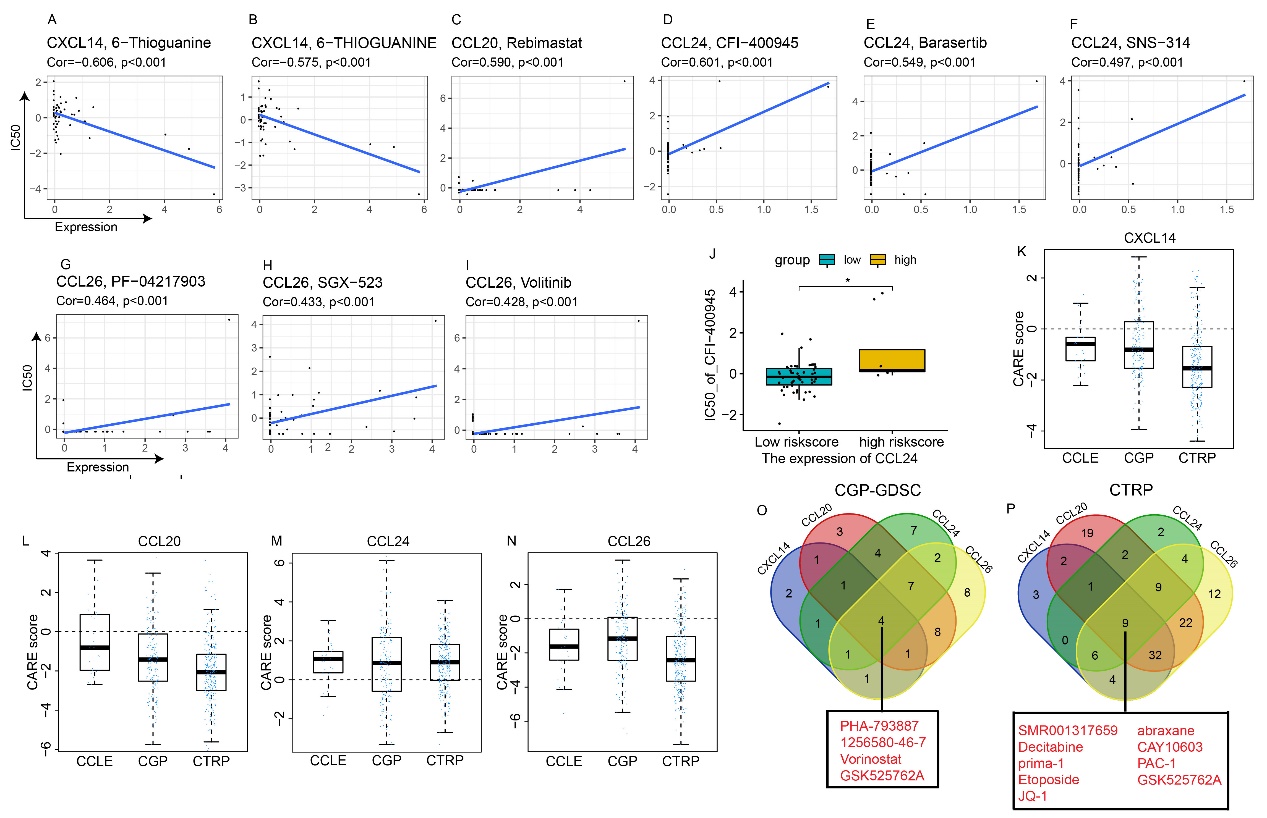


**Supplemental Figure 7. The implications of chemokine genes for drug therapy in PCa.** The results of the CellMiner showed the association between the expression of CXCL14, CCL20, CCL24, and CCL26 and drug sensitivity (**A-J**), The results of CARE indicated the association between the expression levels of CXCL14, CCL20, CCL24, and CCL26 and drug therapy efficacy from the CCLE, GDSC/CGP, and CTRP datasets (**K-N**), and the Ven diagrams showed the common drugs that targeted CXCL14, CCL20, CCL24, and CCL26 (**O-P**).

**
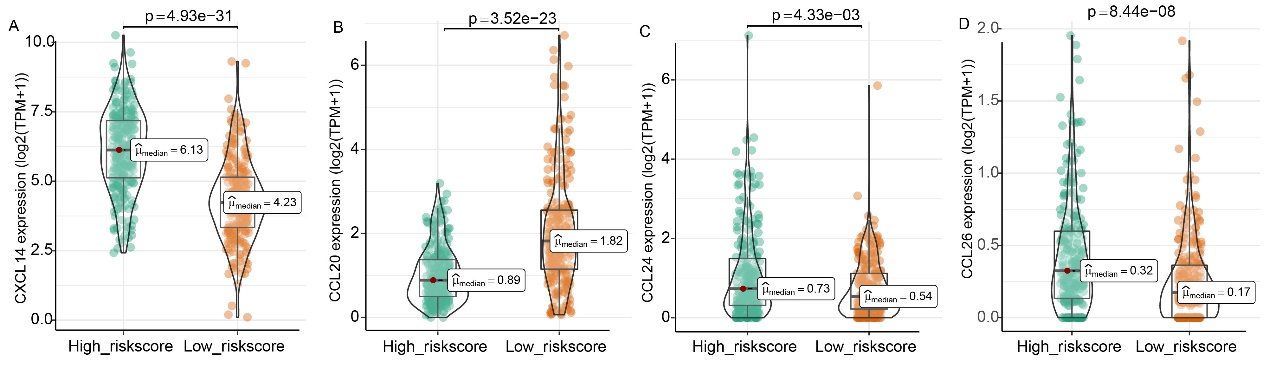
**

**Supplemental Figure8. The distribution of CXCL14, CCL20, CCL24, and CCL26 in high- and low-risk PCa patients (A-D).**
